# Design, synthesis, and biological evaluation of novel imidazole derivatives as analgesic and anti-inflammatory agents: experimental and molecular docking insights

**DOI:** 10.1038/s41598-024-72399-8

**Published:** 2024-10-04

**Authors:** Gulam Muheyuddeen, Mohd Yaqub Khan, Tanzeem Ahmad, Shriyansh Srivastava, Stuti Verma, Mo. Suheb Ansari, Nilanchala Sahu

**Affiliations:** 1 Department of Pharmaceutical Chemistry, Faculty of Pharmacy, Jahangirabad Institute of Technology, Jahangirabad Fort, Jahangirabad, Barabanki, 225203 Uttar Pradesh India; 2 Department of Biomedical Engineering, Chung Yuan Christian University, Taoyuan, Taiwan; 3 Department of Pharmacy, Integral University, Lucknow, Uttar Pradesh India; 4 Department of Pharmacy, School of Medical and Allied Sciences, Galgotias University, Greater Noida, Uttar Pradesh India; 5 Department of Pharmacology, Delhi Pharmaceutical Sciences and Research University (DPSRU), Sector 3 Pushp Vihar, New Delhi, 110017 India; 6 Department of Pharmacy, Aryakul College of Pharmacy and Research, Sitapur Village, Jajjaur, Post, Manawa (Near Krishi Vigyan Kendra Sitapur) Sidhauli, Sitapur, Uttar Pradesh India; 7 Sharda School of Pharmacy, Sharda University, Greater Noida, 201310 Uttar Pradesh India

**Keywords:** Imidazole derivatives, Schiff base synthesis, Anti-inflammatory agents, Analgesic activity, Molecular docking studies, COX-2 inhibition, Diclofenac sodium, Drug screening, Medicinal chemistry, Pharmaceutics, Biochemistry, Chemical biology, Drug discovery, Biomarkers, Medical research

## Abstract

Imidazole moieties exhibit a broad range of biological activities, including analgesic, anti-depressant, anticancer, anti-fungal, anti-tubercular, anti-inflammatory, antimicrobial, antiviral, and antifungal properties. In this study, we explored the use of Schiff base for the synthesis of new imidazole derivatives as anti-inflammatory and pain-relieving agents. A series of eight novel imidazole analogues (2a–h) were prepared in three steps with excellent yields. All compounds were characterized using IR, NMR, and mass spectral data. Their analgesic and anti-inflammatory activities were evaluated using hot plate and paw oedema methods. Compound 2 g (1-(2,3-dichlorophenyl)-2-(3-nitrophenyl)-4,5-diphenyl-1H-imidazole) showed significant analgesic activity (89% at 100 mg/kg b.w.), while compounds 2a (2-(2,6-dichlorophenyl)-1-(4-ethoxyphenyl)-4,5-diphenyl-1H-imidazole) and 2b (2-(2,3-dichlorophenyl)-1-(2-chlorophenyl)-4,5-diphenyl-1H-imidazole) exhibited good anti-inflammatory activity (100% at 100 mg/kg b.w.), comparable to diclofenac salt (100% at 50 mg/kg b.w.). Molecular docking studies were conducted using Schrödinger software version 2021-2, employing the OPLS4 force field for both receptor and ligand preparation. The results were visualized using molecular visualization software such as PyMOL. These studies revealed that compound 2g exhibited the highest binding affinity with the COX-2 receptor (−5.516 kcal/mol). Compound 2g formed three conventional hydrogen bonds with residues GLN-242 (bond length: 2.3 Å) and ARG-343 (bond lengths: 2.2 Å & 2.4 Å). This binding affinity was comparable to that of Diclofenac salt, which showed the highest binding affinity of −5.627 kcal/mol with the COX-2 receptor. Diclofenac salt formed two conventional hydrogen bonds with the residues ARG-344 (bond length: 2.0 Å) and TRP-140 (bond length: 1.7 Å). Later, molecular dynamic simulations confirmed the stable binding affinity of compound 2g with the protein. Furthermore, other compounds also demonstrated potential binding to the receptor-binding pocket region. The anti-inflammatory potential of the synthesized compounds was evaluated using the carrageenan-induced rat hind paw oedema model, while the analgesic potential was assessed using the hot plate method. These evaluations were conducted in comparison with Diclofenac sodium, serving as the standard compound. However, compound 2g stood out for its superior analgesic activity, as confirmed by in-vivo examination. These findings suggest that these novel imidazole derivatives have potential as anti-inflammatory and analgesic agents.

## Introduction

Pain is an uncomfortable sensory and emotional experience correlated to real or potential tissue damage, or described as such injury^1^. Pain is always an anecdotal observation. It is without a doubt a sensation in one or more body parts, but it is also invariably unpleasant, making it a psychological experience. About relieving pain for cancer patients, the Organization (WHO) proposed standings for analgesics in 1986. A significant health project, the World Health Organization's Initiative for Cancer Pain and Palliative Healthcare, which included the analgesic ladder, aimed to improve pain alleviation methods through academic executives, the creation of approaches, and the creation of a worldwide support network. Analgesics are drugs that selectively alleviate pain by affecting peripheral and CNS pain mediators without impairing perception^2,3^. You can use a narcotic or non-narcotic analgesic. Research on animal suffering raises ethical, philosophical, and practical concerns. Inflammation is a retort of the nonspecific immune system which obliges to localise, neutralise, or decimate a deleterious agent to prepare for the rehabilitation process. It is a local reaction of the endothelial and aiding elements of a tissue to the concussion that results in the formation of protein-rich exudates. It is a defence mechanism of the generalized immune response that helps to localize, neutralize, or destroy an injurious agent in preparation for the method of rehabilitation.

Inflammation is the predominant way of responding to a pathogen or injurious stimuli. This response includes the release of pro-inflammatory chemicals as a consequence, these chemicals increase blood vessel permeability and cause leakage of fluid into the tissues that results in pain, heat, redness, swelling, and loss of function^4^.The main two types of inflammation are acute and chronic inflammation^5^. Medications that are commonly used to treat inflammation are nonsteroidal anti-inflammatory drugs (NSAID) and corticosteroids. The latter is known to cause both local and systemic side effects^6^. Moreover, several undesirable adverse effects could be present due to the chronic usage of nonsteroidal anti-inflammatory drugs (NSAID)^7^. Thus, in the pharmaceutical industry, imidazole is being investigated and the substituted forms of imidazole have also been used in various therapeutic applications. Several effective drugs are currently on the market and some of them are in clinical trials at different phases.

In investigating imidazole derivatives, the current available studies reported promising anti-inflammatory activity based on their mechanism of action and important Structure–activity relationship.

The swelling is an immediate reaction of the vascular and other supportive elements of a tissue after damage that results in the generation of a protein-rich transudes. Rubor (redness), calor (heat), dolor (pain), tumor (swelling), and functio laesa (loss of function) are the five cardinal hallmarks of inflammatory conditions. Aetiology of inflammatory processes encompasses physical entities, chemical substances, triggering immune responses, and contagion by infectious agents. Two categories of inflammation exist: acute and chronic. Acute inflammation is characterized by the exuding of fluid and plasma proteins (oedema) with the evacuation of leukocytes, particularly neutrophils. The chronic inflammatory response is seen as inflammation that lasts for a long time (weeks or months), during which time there is concurrent aggressive tissue damage, inflammation, and endeavours at healing. Numerous of the most prevalent and incapacitating pathological conditions, including anthropathy; atherosclerosis; phthisis; and chronic lung diseases; are characterised by chronic inflammation^8^. Nonsteroidal anti-inflammatory drugs (NSAIDs) are frequently prescribed as the first line of therapy for a range of autoimmune disorders such as arthritis and rheumatism along with treating minor aches and pains associated with daily life^9^. Conventional NSAIDs work by suppressing PG production, a few of them being pro-inflammatory. Restricting the rate-limiting COX enzyme connected to the inflammatory cascade is the main way to achieve this^10^. Imidazole and fused imidazole with six-membered rings^11^ are the most broadly adopted compounds as analgesic and anti-inflammatory drugs among the various forms of NSAIDs. Because of their crucial features as medicines in new therapies, fused imidazole analogues have taken a major position in medical research^12^. As a result, the pharmaceutical industry is researching imidazole and a variety of medicinal uses for substituted imidazole analogues are being discovered^13,14^. A wide range of biological actions are present in imidazole analogues due to the adaptable nucleus that is present in many of these compounds^15,16^. Several biological processes, including antiarrhythmic, HIV-RT inhibitor^17^, agrochemicals, antiulcer, anti-inflammatory, vermicidal, inotropic, anti-allergic, Anti-microbial, anti-viral, and cytotoxicity^18,19^ have been associated with it, making it a particularly significant pharmacophore and privileged configuration in drug design^20,21^. Consequently, the development of several powerful medications, including Omeprazole (PPI), Pimobendan (an ionodilator), Albendazole (an inhibitor of encephalitozoon intestinalis infection in AIDS patients), and mebendazole (anthelmintic) has been made possible by the optimization of imidazole analogue based on their structural characteristics. Synthesis of the Imidazole analogue has indeed garnered a lot of attention in subsequent decades.

Heterocyclic compounds have ring structures that include one element rather than carbon atoms, like as sulphur, nitrogen or oxygen. They occur naturally in a variety of substances, including nucleic acids, plant alkaloids, anthocyanin’s, and flavonoids^22^. They are the side groups of DNA and RNA, the most common and fundamental components of living cells, and they are extremely important in biochemical activities. They are essential for all living cells' metabolism, as well. There is a large variety of heterocyclic compounds that are pharmacologically active, many of which are used often in clinical environments. Most medications introduced in pharmacophore in recent years have been made of heterocyclic compounds^23,24^. Studies have shown that particular structural groups of heterocyclic compounds have biological functions. Among the most crucial synthetic techniques for finding new drugs is the imidazole moiety. Therapeutic medications now include a wider range of imidazole analogues, including those with anti-inflammatory; anti-diabetic; anticoagulant, anti-cancer, antibacterial; antifungal; antiviral; antitubercular and antimalarial properties^25,26^. Cimetidine (an antihistaminic), metronidazole (an anti-parasitic), histidine (an important amino acid), dacarbazine (an anti-tumoral, anti-cancer drug), and losartan (a blood pressure medication) are among the bioactive substances that include the imidazole nucleus (antihypertensive)^27,28^. 1, 3-diaza-2, 4-cyclopentadiene derivatives, and pharmacologic actions against anticancer, antiviral, antibacterial, & antifungal strains were demonstrated for the numerous heterocyclic generated from them^29,30^.

**Fig. 1:**
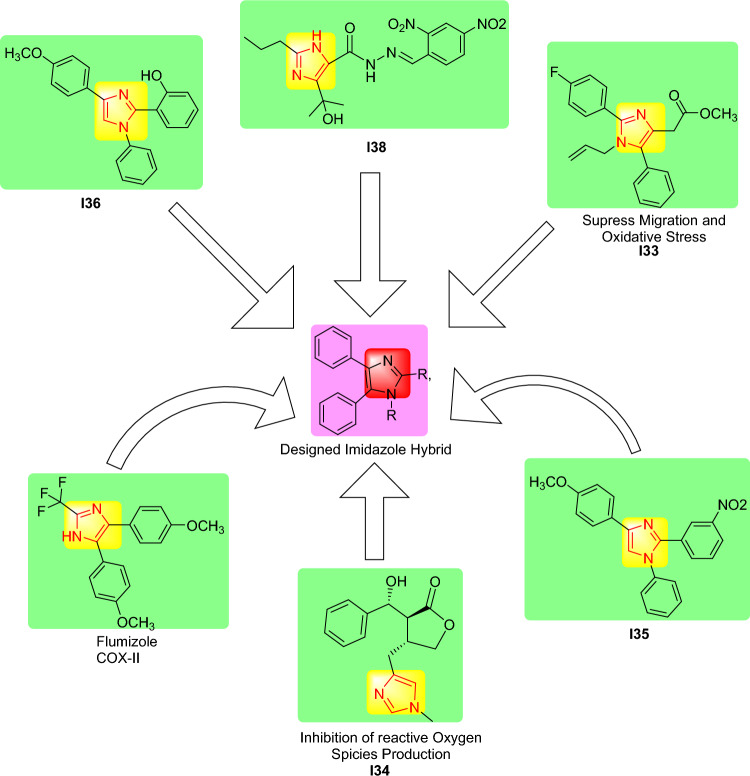
Rational drug design approach used to prepare imidazole hybrids.

Nascimento et al. have synthesized eight novel imidazole derivatives to be tested as a potential anti-inflammatory agent in vitro. Compound I33 was the most potent inhibitor of nuclear factor kappa B (NF-κB) transcription factor transmigration. Furthermore, compound I33 (showing in Fig. 1) effectively reduced the pro-inflammatory mediators in addition to inhibition of nitric oxide release in J774 macrophage. The author concluded that compound I33 demonstrated similar efficacy in both in vitro and in vivo studies suggesting it could serve as a potential anti-inflammatory agent^31^. In this regard, Rocha et al. have investigated two imidazole alkaloids to study the anti-inflammatory activity on human neutrophils with Indomethacin as a reference agent. The results of this study showed that I34 (showing in Fig. 1) has good activity against neutrophil degranulation, reactive oxygen species (ROS), and IL-6 production. These results were confirmed using an in vivo model that decreased inflammatory hyper nociception and myeloperoxidase (MPO) release in mice^32^.

Moreover, some authors reported SAR studies based on applying a medicinal chemistry approach from available anti-inflammatory drugs. For example, Husain et al. believed that safe and effective NSAIDs would still be needed. The author explored the potential of di- and tri-substituted imidazole derivatives as anti-inflammatory agents, employing the carrageenan-induced rat paw edema model as a research tool. The study results showed that six compounds displayed remarkable anti-inflammatory activity from 49.58 to 58.02% oedema inhibition with minimal GI discomfort and severity index from 0.17 to 0.34 in vivo. Husain et al. noted that compounds I35 and I36 (showing in Fig. 1) are biologically active lead compounds with promising anti-inflammatory activity that could be utilized as a novel anti-inflammatory candidate^33^.

An additional study in which authors evaluated twenty- three compounds against human erythrocytes. Among the synthesized compounds, compound I38 demonstrated excellent activity with an IC50 of 44 μM which is higher than standard Aspirin (IC50 of 200 μM) and Indomethacin (IC50 of 112 μM). Furthermore, as the number of electrons withdrawing moiety in the benzene ring increases the anti-inflammatory activity also increases. The authors concluded that compound I38 (showing in Fig. 1) with two NO_2_ substitutions on the benzene ring exhibited excellent anti-inflammatory activity compared to aspirin and indomethacin^34^.

Husain et al., 2016, Kaur and Silakari 2017, Lucarini et al., 2020, and Husain et al., 2021 reported that Imidazole is a common structural feature of the anti-inflammatory molecule flumizole. Thus, we contemplated that imidazole ring-containing compounds could impart anti-inflammatory activity to the synthesized compounds. Furthermore, it was hypothesized that clubbing imidazole with indole moiety, a pharmacophore of NSAID indomethacin and many other clinically useful drugs, through a suitable linker in one molecule would yield potent and safer NSAIDs^35–38^.

The diverse pharmacological effects of imidazoles described above motivated researchers to further research the analgesic and anti-inflammatory properties of certain significant imidazole analogues in vivo. Due to the significance of this idea to continue our continuing study on imidazole analogues^39,40^, researchers are eager to investigate an easy & innovative method for making 1, 3-diaza-2, 4-cyclopentadiene derivatives using ingredients that are easily accessible^41,42^ just like substituted amines and benzaldehydes, as well as in-vivo testing of the resulting chemicals for analgesic & anti-inflammatory properties.

### Chemistry

**Fig. 2:**
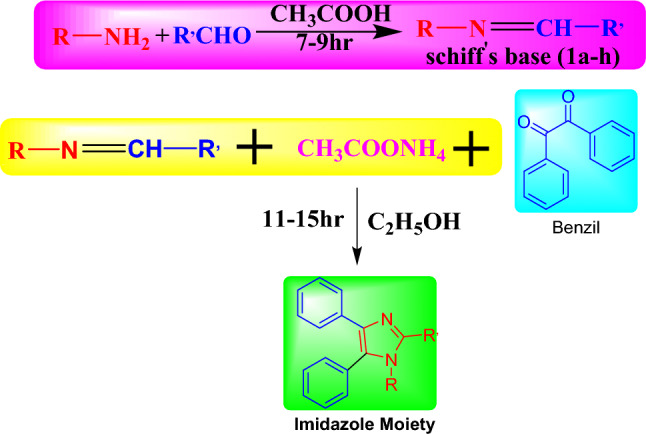
Schematic representation of 1, 3-diaza-2, 4-cyclopentadiene derivatives (2a–h).

Imidazole Derivatives are versatile nitrogen-containing heterocyclic compounds that have a long history of being considered as a potential class of biologically active substances with various therapeutic actions, like as anti-inflammatory analgesic, antimicrobial, anticancer, antifungal, anti-tubercular, anti-convulsant, and anthelmintic. Because of the medicinal importance of the Imidazole nucleus fused with pyrazole as a potential therapeutic agent, it was thought worthwhile to synthesize and characterise certain newer compounds having Imidazole nuclei and evaluate their biological potential. To get novel products with outstanding yield as stated in (Fig. 2 is the scheme), the synthetic technique was investigated^43,44^. In a small evaporating dish, 20 ml of glacial acetic acid was mixed with an equal quantity (0.01 M) of substituted aromatic amine and substituted benzaldehydes. The mixture was heated in a water bath for 7–9 h while being constantly stirred. After the reaction was complete, the mixture was then quenched in cold water and stirred again^15^. The derivatives was recrystallized via ethanol after solidification to produce Schiff's base (substituted). In Table 1, melting points, yield, and elemental analyses were provided. The versatility of imidazole derivatives makes them extremely valuable, as their structure can be modified to produce a wide range of compounds with diverse properties and activities, allowing for the development of novel therapeutic agents with optimized efficacy and selectivity. The produced products were subsequently evaluated by elemental analysis, IR, NMR, ^1^H and ^13^C.

**Table 1:** Physical data of synthesized compound.

Compound	R	R’	Chemical formula	Isolated yield^a^ (%)	Melting point $({^\circ{\rm C} }$)	H		O		C	
						Calculated	Findings	Calculated	Findings	Calculated	Findings
**2a**	Cl	OEt	C_29_H_22_C_l2_N_2_O	60	198–200	75.31	75.37	5.87	5.79	18.82	18.68
**2b**	Cl	Cl	C_27_H_17_C_l3_N_2_	58	280–282	65.25	65.05	4.69	4.54	16.30	16.17
**2c**	Cl	NO_2_	C_27_H_18_ClN_3_O_2_	74	240–242	55.65	55.46	4.00	3.86	13.91	13.78
**2d**	Cl	F	C_27_H_17_C_l2_FN_2_	63	165–167	71.13	71.07	5.97	5.99	16.59	16.45
**2e**	Cl	OMe	C_28_H_20_C_l2_N_2_O	55	236–238	55.65	55.60	4.10	4.00	13.91	13.79
**2f**	Cl	Br	C_27_H_18_BrClN_2_	72	218–220	49.95	49.85	3.29	3.15	12.48	12.35
**2g**	CH3	Cl	C_27_H_17_C_l2_N_3_O_2_	77	210–212	44.13	44.02	2.91	2.82	11.03	10.06
**2h**	NO2	OMe	C_27_H_24_N_2_O	55	286–288	54.23	54.14	4.25	4.16	12.65	12.51

^a^Isolated yield.

### In-silico molecular docking studies with cyclooxygenase-2

Molecular docking calculations were completed using Schrodinger^®^ docking suite (Schrödinger Maestro, Version 12.8, Release 2021-2, Platform Windows- × 64)^45^. This work utilized standard precision SP. Protein(4PH9) was obtained from the Protein Data bank (PDB, https://www.rcsb.org/) and prepared by restrained minimization using force field OPLS4. The grid sites were created using a Glide^®^ receptor grid generator with a docking length of 20 Å. Grid centres were determined from active sites on the target protein. The compounds were obtained from Chem Draw 21.0.0 (https://chemdrawdirect.perkinelmer.cloud/)^46^ in the form of an SDF file with the help of the IUPAC name to structure and standard Diclofenac for comparison was obtained from the PubChem database (https://pubchem.ncbi.nlm.nih.gov)^47^ and visualized with the help of Discovery Studio BIOVIA 2024 (https://discover.3ds.com/)^46^. Ligands were prepared using force field OPLS4 and possible states were generated from pH 7.0 ± 2.0. The possibility of spontaneous binding between the receptor and ligand was demonstrated by a negative docking score (in kcal/mol) the more negative the number, the better binding. Good binding activity between the receptor and ligand was shown by a docking score of less than −4 kcal/mol. All the docking results were visualized by the Schrodinger molecular viewer by ligand interaction for 2D diagrams and compounds exhibiting the conventional hydrogen bond(s) as an interaction with the receptor’s binding pocket region were visualized by the PyMOL molecular visualizer (https://pymol.org/2/) for getting 3D diagrams^48^.

#### Molecular dynamic simulation

The simulation study was performed to determine the stability of the docked protein–ligand complexes using the automated SiBioLead web server (https://sibiolead.com/) that employs the GROMACS package for simulation. The molecular simulation with SiBioLead generally runs with the basic steps of data preparation, pre-processing, energy minimization, equilibration, molecular dynamics, trajectory analysis and result generation. In data preparation, the docked complex files in (.pdb) format were downloaded from the Schrödinger Maestro. Subsequently, in pre-processing, the complex file format was uploaded and checked to the broken residues through its automatic GROMACS preprocessing system also, selected on keeping all ligands, the name of the attached residue of the docked ligand was provided (which was obtained from Discovery Studio BIOVIA 2024). Then the MMPBSA analysis was initiated. The preprocessing parameters was set at AMBERSS9B forcefield, water model at simple point charge (SPC), cubic box type and with neutralizing salt NaCl at concentration of 0.15 molar. In the energy minimization process, the integrator was set at the steepest descent with 5000 steps. In equilibration process, the type of equilibration was set at NVT/NPT with 100 ps of equilibration time at a simulation temperature of 300 K and simulation pressure of 1 bar. In simulation process, the leap frog simulation integrator was used with 1 ns simulation time and 1000 frames. Finally, in simulation analysis, the results were obtained as root mean square deviation (RMSD) of the backbone and the ligand, root mean square fluctuation (RMSF), radius of gyration (Rg), binding free energy of the target ligand (MMPBSA) and H-Bond estimation^49^.

### Pharmacological assessment

All of the synthesized compounds were examined for their pharmacological properties, including their analgesic and anti-inflammatory effects within living organisms. The physiological reactions of animals to heat & chemical triggers have been measured during such endeavours. Mice were used in the eddy hot technique for 50 mg/kg of b.w. has painkiller along with soothing actions^50,51^. Comparing the degree of safeguarding to the common medication diclofenac sodium, a calculation was made. When evaluated to diclofenac sodium, all substances demonstrated strong efficacy in investigating its soothing properties, carrageenan-persuaded inflammation on rat rear paw oedema techniques has been adopted^52^, in mice at 50 mg/kg b.w. were acted upon. To calculate the percent inhibitory effect for synthesised analogues, the commonly recommended diclofenac sodium as standard was used.

## Result and discussion

### Chemistry

As the scheme was prepared after the literature review the synthesis of Imidazole derivatives was decided to be made in two different steps at the initial step substituted amines were reacted with substituted benzaldehydes to form Schiff base using glacial acetic acid. Table 2 contains Substituted Amine and substituted benzaldehydes on R and R’ positions**.** Further Schiff base is treated with ammonium acetate and benzil in the presence of ethanol and refluxed for 12 h (Fig. 2) to give desired compounds of Imidazole derivatives **(2a–h).** TLC, IR, NMR, melting point and solubility performed the conformation of the structure of the compounds.

**Table 2:** Substituted on R and R’ position.

Compound	R	R’
2a	3,4-Dichloroaniline	P-Ethoxy benzaldehyde
2b	2,3-Dichloroaniline	2-Chloro benzaldehyde
2c	P-Chloroaniline	2-Nitro benzaldehyde
2d	2,3-Dichloroaniline	4-Fluoro benzaldehyde
2e	2,3-Dichloroaniline	3-Methoxy benzaldehyde
2f	2-Chloroaniline	2-Bromo benzaldehyde
2g	3-Nitroaniline	2,3-Dichloro benzaldehyde
2h	P-Toluidine	P-Methoxy benzaldehyde

IR spectra of Imidazole derivative **(2a–h)** showed a peak between 3020 and 3088 cm^−1^ confirming the presence of aromatic –CH stretching. The region of 1523–1598 cm^−1^ due to C=C stretching. They showed peaks between 1621 and 1670 cm^–1^ of the C=N stretch indicating the presence of C=N in the Imidazole nucleus. In ^1^H NMR spectrum shows Singlet value –OCH_3_ is 3.70. The C=CH_3_ protons of methylene appeared at 1.97. The aromatic protons show their appearance between 6.38 and 7.95.

### Molecular docking analysis

**Fig. 3:**
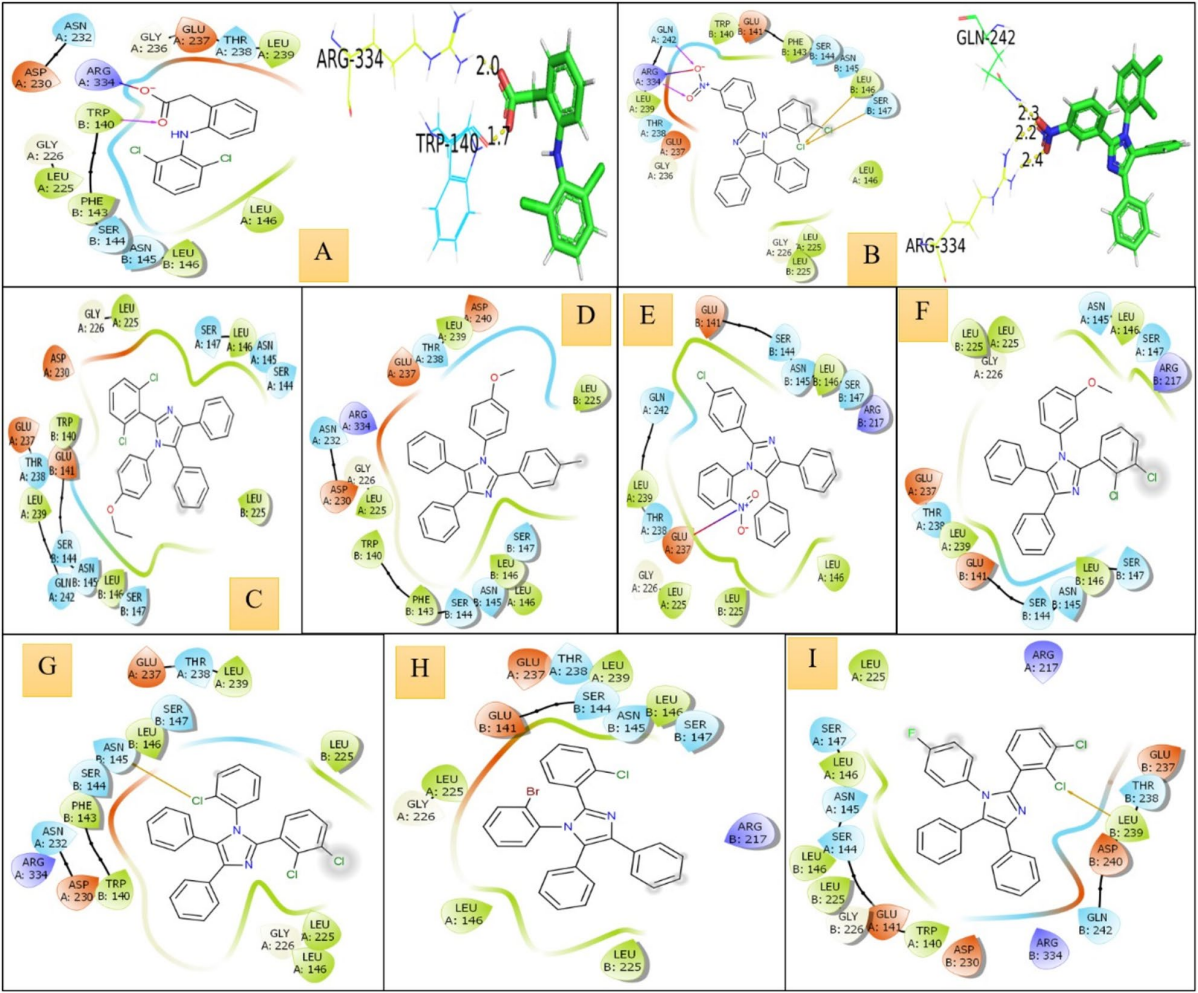
Molecular docking analysis results of COX-2 with different compounds obtained from Schrodinger molecular visualizer (2D diagrams) and PyMOL for 3D diagrams [(**A**) diclofenac-standard, (**B**) 2g, (**C**) 2a, (**D**) 2h, (**E**) 2c, (**F**) 2e, (**G**) 2b, (**H**) 2f, (**I**) 2d].

**Fig. 4:**
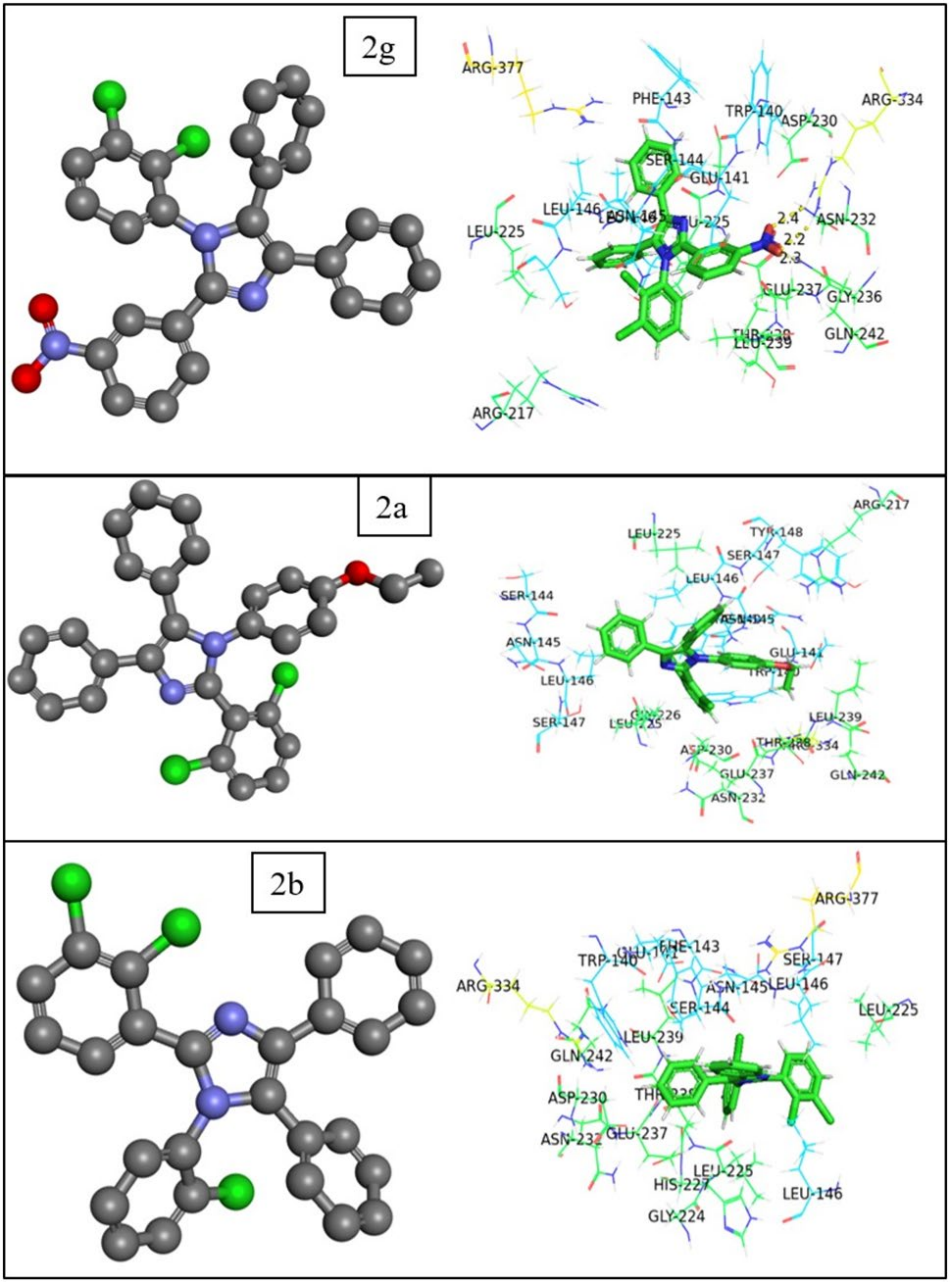
Molecular docking analysis results of COX-2 with different compounds obtained PyMOL for 3D diagrams of potent compounds (B: 2g; C: 2a; G: 2b).

PDB ID selected for target COX-2 was 4PH9 for performing molecular docking analysis with all the compounds and then compared with a standard control drug Diclofenac. Among all the docking complexes formed, Diclofenac showed the highest binding affinity of −5.627 kcal/mol with the COX-2 receptor by two conventional hydrogen bonds to the residues ARG-344 (bond length: 2.0 Å) and TRP-140 (bond length:1.7 Å). The compound that exhibited the highest binding affinity with the COX-2 receptor was 2g, with a binding energy of −5.516 kcal/mol by three conventional hydrogen bonds to the residues GLN-242 (bond length: 2.3 Å) and ARG-343 (bond lengths: 2.2 & 2.4 Å). The compounds 2a, 2h, 2c, 2e, 2b, 2f and 2d were bound to the receptor with a binding energy of −4.478 kcal/mol, −4.411 kcal/mol, −4.348 kcal/mol, −4.216 kcal/mol, −4.181 kcal/mol, −3.969 kcal/mol and −3.891 kcal/mol respectively, without forming any conventional hydrogen bond to the bonding pocket residues. The docking pose of all the compounds revealed significant interaction with the active site of the respective targets. Moreover, potential hydrophobic contacts were found at the active site of the receptors. The significant docking score values imply that these compounds could represent potential leads for future non-steroidal anti-inflammatory drugs (NSAIDs). The protein–ligand interaction of the compounds is visualized in Figs. 3 and 4. Supplementary Table 1 presents the 2D and 3D representations of the molecular docking results, illustrating the binding interactions and conformations of the docked molecules.

### Molecular dynamic simulation

**Fig. 5:**
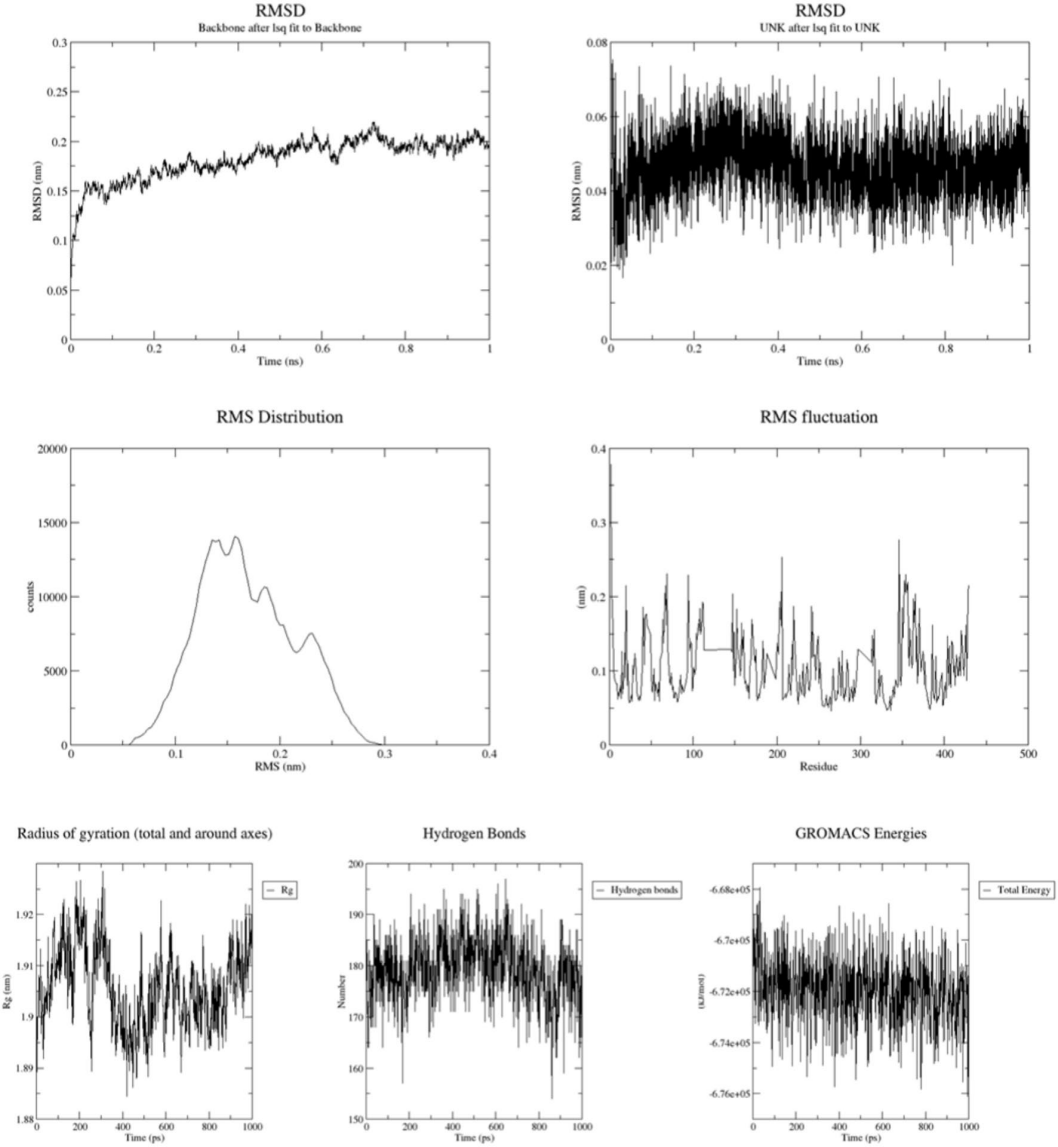
Simulation parameters for 4PH9 and 2g compound-complex.

The docked complex with the highest docking score (4PH9 and compound 2g) was subjected to molecular dynamics simulations using SiBioLead to evaluate and compare its stability (Fig. 5). The analysis focused on the binding stability of compound 2g to protein 4PH9. The results indicated identical RMSD values (nm) for the backbone, nearly identical RMSD values for the ligand, and similar ranges for RMSF, Rg, H-bond counts, and MMPBS binding free energy. Specifically, the RMSD (nm) of the backbone ranged from 0.1 to 0.2, the RMSD (nm) of the ligand ranged from 0.02 to 0.06, major RMSD peaks were observed between 10,000 and 15,000 counts at 0.1 to 0.2 nm, with a minor peak between 5000 and 10,000 counts at 0.2 to 0.3 nm. The RMSF (nm) for 500 residues ranged from 0.05 to 0.2, the Rg (nm) over 1000 ps ranged from 1.89 to 1.92, the number of H-bonds over 1000 ps ranged from 170 to 190, and the MMPBS binding free energy (KJ/mol over 1000 ps) was approximately −6.72e+05.

### Analgesic activity

Using the eddy hot plate method, the synthesised compounds' analgesic efficacy was evaluated. Structure–activity relationship (SAR) analyses reveal that virtually all of the analogues exhibit extremely high analgesic action when juxtaposed with the medicine diclofenac sodium. Among the tested compounds 1-(2, 3-dichlorophenyl)-2-(3-nitrophenyl)-4,5-diphenyl-1H-imidazole (2g) demonstrated clear analgesic effects (89%, 100 mg/kg b.w).

**Fig. 6:**
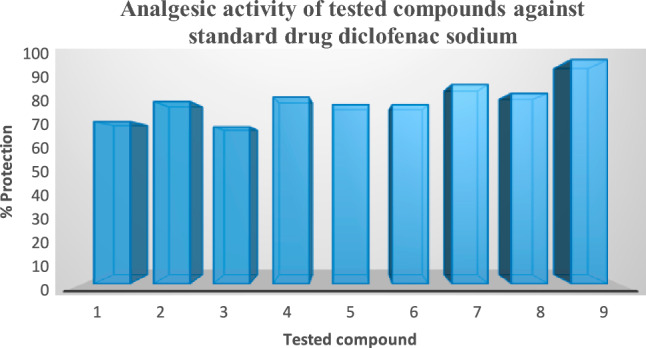
Analgesic effect of tested analogues at (100 mg/kg b.w) and diclofenac sodium (50 mg/kg b.w). Concentration has been analysed.

Due to the addition of bromo, chloro, methoxy, fluoro, nitro, and ethoxy-substituted aryl *N*-(4-ethoxybenzylidene)-2,6-dichlorobenzenamine-substituted Schiff bases to chloro and nitro substituted aniline, all of the further analogues such as 2a, 2b, 2c, 2d, 2e, 2f, and 2h have also demonstrated significant activities. It has been demonstrated that adding bromine & the nitro group to substituted aniline and bromo, chloro, methoxy, and ethoxy to substituted benzaldehydes at various locations increased the analgesic action inside the same ring framework, i.e., 1-(2-bromophenyl)-2-(2,3-dichlorophenyl) (Table 3 and Fig. 6) contain summaries of the findings.

**Table 3:** Analgesic effect of tested analogues at (100 mg/kg b.w) and Diclofenac sodium (50 mg/kg b.w). Concentration have been analysed.

Compound	Mean writhing (X ± SE)	Protection
2a	5.0 ± 2.08**	72.33
2b	**8.3** ± 3.33**	**81.33**
2c	5.6 ± 1.85**	70.00
2d	8.3 ± 1.45**	**83.33**
2e	5.0 ± 2.51**	**80.00**
2f	6.0 ± 0.57**	**80.00**
2g	3.3 ± 1.66**	**89.00**
2h	4.3 ± 2.02**	**85.00**
Control	30.0 ± 1.55**	–
Standard	–	100.00

The information is shown as the final value for each group (Diclofenac sodium & investigated substances) over 3 h & represents the average values SE of 3 mice per batch.

Data had been analysed by one-way ANOVA followed by Turkey–Krammer Multiple comparison tests **p < 0.05.

Baseline (pre-drug) values and post-drug values were used to figure out the percentage alteration. Protection was figured out as concerns to the percentage alteration of the diclofenac sodium.

*SE* standard error, *Diclo*. diclofenac sodium.

Bold type denotes the active substances.

### Anti-inflammatory activity

**Fig. 7:**
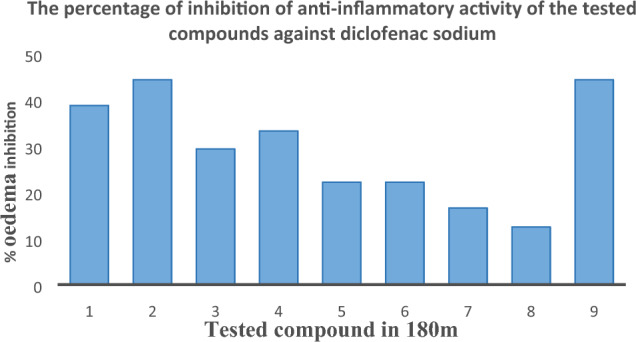
% inhibition of the soothing effect of the investigated agents (100 mg/kg, b.w) and diclofenac (50 mg/kg, b.w) in 180 m.

**Fig. 8:**
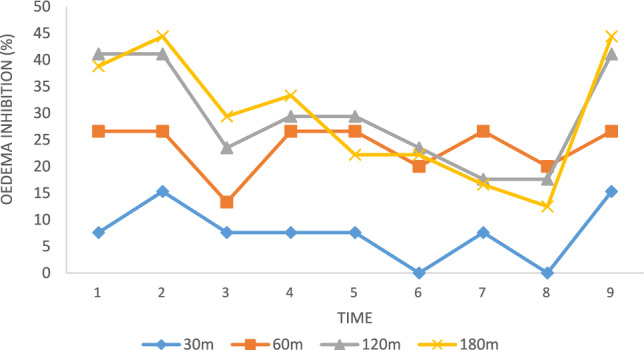
The inhibition of the soothing effect of the investigated agents (100 mg/kg, b.w) and diclofenac (50 mg/kg, b.w).

Before administering the carrageenan, the outcomes among the evaluated molecules with the reference standard had been evaluated. Inflammation within rats caused by the injection of carrageenan was assessed at intervals of 30, 60, 120, and 180 min. The per cent suppression of oedema was computed with the saline control group, as illustrated in Table 4, as well as Figs. 3 and 4. When compared to the standard medicine diclofenac sodium, the majority of the examined substances have produced positive outcomes. Analogues (2a) and (2b) exhibit the strongest anti-inflammatory effects of all the chemicals. If compared with various substituted benzaldehydes (–Br and –NO_2_, etc.) with the conventional medication diclofenac sodium, the chloro-substituted aniline and p-toluidine have demonstrated significant soothing effects. The synthesis of a novel category of imidazole analogues has been given a fresh direction as the outcome (Table 4, Figs. 7 and 8) is a summary of the findings.

**Table 4:** The soothing effect of the investigated agents (100 mg/kg, b.w) and diclofenac (50 mg/kg, b.w).

Compound	Paw oedema thickness (nm)							
	30 m (X ± SE)	% Oedema inhibition	60 m (X ± SE)	% Oedema inhibition	120 m (X ± SE)	% Oedema inhibition	180 m (X ± SE)	% Oedema inhibition
Control	1.3 ± 0.05	**–**	1.5 ± 0.03	**–**	1.7 ± 0.03	**–**	1.8 ± 0.03	**–**
**2a**	1.2 ± 0.03	**7.6**	1.1 ± 0.00**	**26.6**	1.1 ± 0.03**	**41.1**	1.1 ± 0.05**	**38.8**
**2b**	1.1 ± 0.03	**15.3**	1.1 ± 0.00**	**26.6**	1.1 ± 0.03**	**41.1**	1.0 ± 0.03**	**44.4**
**2c**	1.2 ± 0.05	7.6	1.3 ± 0.03**	13.3	1.3 ± 0.03**	23.5	1.3 ± 0.06**	**29.4**
**2d**	1.2 ± 0.06	7.6	1.1 ± 0.05**	**26.6**	1.2 ± 0.03**	**29.4**	1.2 ± 0.06**	**33.3**
**2e**	1.2 ± 0.03	7.6	1.1 ± 0.03**	**26.6**	1.2 ± 0.05**	**29.4**	1.4 ± 0.05**	**22.2**
**2f**	1.3 ± 0.05	**–**	1.2 ± 0.05**	20.0	1.3 ± 0.08**	23.5	1.4 ± 0.05**	**22.2**
**2g**	1.2 ± 0.03	7.6	1.1 ± 0.06**	**26.6**	1.4 ± 0.03**	17.6	1.5 ± 0.05**	16.6
**2h**	1.4 ± 0.00	**–**	1.2 ± 0.03**	20.0	1.3 ± 0.10**	23.5	1.5 ± 0.05**	16.6
Diclofenac	1.1 ± 0.05	15.3	1.1 ± 0.00**	26.6	1.0 ± 0.00**	41.1	1.1 ± 0.00**	**44.4**

The data show the percentage variations at 30, 60, 120, and 180 m after “carrageenan injection”, as well as the mean value ± SE of six mice per batch.

One-way ANOVA was used to evaluate the data, and then the “Turkey–Krammer Multiple Comparison Analysis” **p < 0.05 was performed.

According to the saline control group, the percentage of oedema inhibition was computed.

**Significant difference from the control value at p < 0.05.

*SE* standard error, *Diclo* diclofenac sodium.

The words "active compounds" are bolded.

## Conclusion

In conclusion, we have successfully developed an efficient and straightforward pathway for synthesizing 1-(2,3-dichlorophenyl)-2-(3-nitrophenyl)-4,5-diphenyl-1H-imidazole compounds with high yields. Molecular docking studies revealed that all compounds exhibit potential analgesic properties by binding to and inhibiting COX-2, with compound 2g showing the highest binding affinity and analgesic effect. Molecular dynamic simulation result for 2g compound and targeted protein showed validated and stable binding for molecular docking result for 2g-COX2 complex. In-vivo assays confirmed that all synthesized compounds possess analgesic and anti-inflammatory activities, with analogues 2g, 2a, and 2b exhibiting the highest analgesic and anti-inflammatory effects, respectively. Notably, compounds 2c, 2d, 2e, 2f, and 2h showed significant analgesic effects comparable to diclofenac sodium, and modest anti-inflammatory effects. The chloro substitution in the ortho- and meta-directions of the aryl imidazole fragments enhances the analgesic and anti-inflammatory activities of these analogues, making them potential precursors for the development of effective therapies. These findings suggest that these compounds have the desired pharmacological effects, which will encourage further research in medicinal chemistry to discover analgesic and anti-inflammatory drugs with halogen and –CH3 functional groups. The cytotoxicity studies Table 5 has been performed by the ProTox 3.0 (https://tox-new.charite.de/).%20.

**Table 5:** Cytotoxicity studies of synthesized analogues and standard drug diclofenac sodium.

Compound Name	Hepatotoxicity	Neurotoxicity	Nephrotoxicity	Carcinogenicity	Immunotoxicity	Mutagenicity	Cytotoxicity
2a	−	+	−	+	−	−	−
2b	−	+	−	+	−	−	−
2c	+	+	−	+	−	+	−
2d	+	+	−	+	−	−	−
2e	+	+	−	+	−	−	−
2f	−	+	−	+	−	−	−
2g	+	+	−	+	−	+	−
2h	−	+	−	+	−	−	−
Diclofenac sodium	+	+	+	+	−	−	−

Cytotoxicity is indicated by a positive ( +) sign, while non-toxicity is denoted by a negative (−) sign. A comparative cytotoxicity study was conducted using ProTox 3.0, a specialized software tool, to evaluate the cytotoxicity potential of our synthesized compounds relative to diclofenac sodium. According to the predictions generated by ProTox 3.0.

### Research procedure

#### Assets and approaches

The products' MP $(^\circ \text{C}$) were found to be incorrect when they were measured using open capillaries on Buchi equipment. The infrared spectrum was compiled on a Shimadzu-8400S’s FT-IR Spectrophotometer, using KBr pellets. A Bruker Avance-400/AvIII HD 300 MHz (FT NMR) spectrometer had also been used to analyze ^1^H and ^13^C NMR spectrum in chloroform-d with Tetramethylsilane serving as a standard for internal use. The 1H resonant frequency was 300 M-hertz, while the 13C resonant frequency was 75 M-hertz. To corroborate the assignment of the N–H protons' spectrum, D2O exchange has been employed. Records of the Mass spectrum had been made using a waters Alliance e-2695/HPLC TQ-D mass spectroscopy. The elemental analysis had been finished with Heraus CHN rapid analyzer. All of the substances produced C, H, and N analyses that were in 0.5% of the predicted values. TLC has been employed to analyze the uniformity of the analogues on aluminium silica gel 60 F254 (Merck), which were observed by UV rays (254 nm) along with iodine vapours. Each ingredient was chemically or analytically pure.

### Basic steps for synthesizing 1-(2-bromophenyl)-2-(2,3-dichlorophenyl)4,5-diphenyl-1H-imidazole (2a–h)

A flask containing benzil (0.01) and Schiff's base (0.01 M) was mixed in the existence of extra CH_3_COONH_4_ (0.1 M). It was refluxing the mixture between 12 and 15 h. Further, TLC monitored the process of progress of completion. To remove the acetic acid and ammonium acetate from the reaction mixture, 250 ml of H_2_O was added precipitate was filtered, washed in benzene (230 m), and recrystallized from ethyl acetate to produce the proper imidazole to remove any leftover benzyl^53^. Supplementary Figures 9 and 10 represent the results of spectroscopic analysis.

#### 2-(2,6-Dichlorophenyl)-1-(4-ethoxyphenyl)-4,5-diphenyl-1H-imidazole (2a)

Yield 60%; MP 198–200 ºC; eluent-n- C_6_H_14_:CH_3_OH (8.6:1.6)v/v, Rf value = 0.71; ^1^H NMR (300 MHz, DMSO-d6 ) δ (ppm): 1.77 (s, 3H, –CH_3_) 3.21 (s, 2H, –OCH_2_), 7.36–7.89 (m, 6H, Ar H), 8.07–8.29 (m, 5H, Ar H), 9.12–9.51 (m, 6H, Ar–H).; IR (KBr, υ, cm^−1^): 3022.75 (Ar, C–H str), 3513.22 (Ali, C–H str), 1581.07 (Ar, C=C str), 1647.18 (C=N), 1319.29 (C–N), 1206.43 (C–O–C Asymmetric) 1036.73 (Ar. C–Cl); EIMS; Found, (*m/z*): 484.1 [M + H]^+^. **C**_29_H_22_Cl_2_N_2_O. Calculated, 482.1.

#### 2-(2,3-Dichlorophenyl)-1-(2-chlorophenyl)-4,5-diphenyl-1H-imidazole (2b)

Yield 58%; MP 280–282 ºC; eluent-n- C_6_H_14_:CH_3_OH (8.5:1.;5)v/v, Rf value = 0.61; ^1^H NMR (300 MHz, DMSO-d6 ) δ (ppm): 1.77 (s, 3H, –CH_3_) 3.31 (s, 2H, –OCH_2_ ), 6.54–6.86 (m, 5H, Ar H), 6.92–7.14 (m, 3H, Ar H), 7.13–7.20 (m, 4H, Ar–*H*); IR (KBr, υ, cm^−1^): 3042.8 (Ar, C–H str), 3343.88 (Ali, C–H str), 1594.54 (Ar, C=C str), 1648.34 (C=N), 1336.20 (C–N), 768.75 (C–O–C Asymmetric) 1032.65 (Ar. C–Cl); EIMS; Found, (*m/z*): 474.0 [M + H]^+^. **C**_27_H_17_Cl_3_N_2_. Calculated, 472.0.

#### 2-(4-Chlorophenyl)-1-(2-nitrophenyl)-4,5-diphenyl-1H-imidazole (2c)

Yield 74%; MP 240–242 ºC; eluent-n-C_6_H_14_:CH_3_OH (8.5:1.5) v/v, Rf value = 0.65; ^1^H NMR (300 MHz, DMSO-d6 ) δ (ppm): 6.13–6.43 (m, 6H, Ar H), 6.58–7.02 (d, 5H, Ar H), 7.19.7.57 (m, 7H, Ar–H).; IR (KBr, υ, cm^−1^): 3025.94 (Ar, C–H str), 3313.67 (Ali, C–H str), 1578.44 (Ar, C=C str), 1647.27 (C=N), 1338.24 (C–N), 775.96 (Ar. C–Cl); EIMS; Found, (*m/z*): 451.1 [M + H]^+^. **C**_27_H_18_ClN_3_O_2_. Calculated, 447.1.

#### 2-(2,3-Dichlorophenyl)-1-(4-fluorophenyl)-4,5-diphenyl-1H-imidazole (2d)

Yield 63%; MP 165–167 ºC; eluent-n-C_6_H_14_:CH_3_OH (8.5:1.5) v/v, Rf value = 0.68; ^1^H NMR (300 MHz, DMSO-d6 ) δ (ppm): 6.54–6.86 (m, 7H, Ar H), 7.31–7.54 (m, 3H, Ar H), 7.56–7.76 (m, 7H, Ar–H).; IR (KBr, υ, cm^−1^): 3076.44 (Ar, C–H str), 3557.77 (Ali, C–H str), 1598.17 (Ar, C=C str), 1654.49 (C=N), 1365.59 (C–N), 1032.61 (Ar. C–Cl); EIMS; Found, (*m/z*): 458.1 [M + H]^+^. **C**_27_H_17_Cl_2_FN_2_. Calculated, 455.1.

#### 2-(2,3-Dichlorophenyl)-1-(3-methoxyphenyl)-4,5-diphenyl-1H-imidazole (2e).

Yield 55%; MP 236–238 ºC; eluent-n-C_6_H_14_:CH_3_OH (8.5:1.5) v/v, Rf value = 0.57; ^1^H NMR (300 MHz, DMSO-d6 ) δ (ppm): 3.56 (s, 3H, –OCH_3_), 6.38–6.89 (m, 7H, Ar H), 7.27–7.41(d, 3H, Ar H), 7.54–7.81 (m, 7H, Ar–H).; IR (KBr, υ, cm^−1^): 3044.25 (Ar, C–H str), 3437.68 (Ali, C–H str), 1588.46 (Ar, C=C str), 1384.87(C=N), 1321.76 (C–N),1208.29(C–O–C Asymmetric) 1039.20 (Ar. C–Cl); EIMS; Found, (m/z): 470.1 [M + H]^+^. **C**_28_H_20_Cl_2_N_2_O. Calculated, 468.01*.*

#### 1-(2-Bromophenyl)-2-(2-chlorophenyl)-4,5-diphenyl-1H-Imidazole (2f)

Yield 72%; MP 218–220 ºC; eluent-n-C_6_H_14_:CH_3_OH (8.5:1.5) v/v, Rf value = 0.67; ^1^H NMR (300 MHz, DMSO-d6 ) δ (ppm): 6.61–7.12 (m, 7H, Ar H), 7.34–7.51 (d, 4H, Ar H), 7.64–7.95 (m, 7H, Ar–H).*;* IR (KBr, υ, cm^−1^): 3048.74 (Ar, C–H str), 3518.61(Ali, C–H str), 1584.29 (Ar, C=C str), 1639.37 (C=N), 1328.84 (C–N), 1036.23 (Ar. C–Cl); 547.89(C–Br).; EIMS; Found, (*m/z*): 488.0 [M + H]^+^. **C**_27_H_18_BrClN_2_. Calculated, 484.0*.*

#### 1-(2, 3-Dichlorophenyl)-2-(3-nitrophenyl)-4,5-diphenyl-1H-imidazole(2g)

Yield 77%; MP 210–212 ºC; eluent-n-C_6_H_14_:CH_3_OH (8.5:1.5) v/v, Rf value = 0.77; ^1^H NMR (300 MHz, DMSO-d6 ) δ (ppm): 7.13–7.47 (m, 7H, Ar H), 7.54.7.71 (d, 3H, Ar H), 7.54–7.85 (m, 7H, Ar–H).*;* IR (KBr, υ, cm^−1^): 3054.78 (Ar, C–H str), 3542.87 (Ali, C–H str), 1530.64 (Ar, C=C str), 1671.71 (C=N), 1394.80 (C–N), 1075.75 (Ar. C–Cl).; EIMS; Found, (*m/z*): 485.1 [M + H]^+^. **C**_27_H_17_Cl_2_N_3_O_2_. Calculated, 485.1*.*

#### 1-(4-Methoxyphenyl)-4, 5-diphenyl-2-p-tolyl-1H-imidazole (2h)

Yield 75%; MP 286–288 ºC; eluent-n-C_6_H_14_:CH_3_OH (8.5:1.5) v/v, Rf value = 0.79; ^1^H NMR (300 MHz, DMSO-d6 ) δ (ppm): 1.98 (s, 3H, -CH_3_) 3.13 (s, 3H, –OCH_3_), 6.28–7.53 (m, 4H, Ar H), 7.29–7.41 (d, 7H, Ar H), 7.34–7.79 (m, 7H, Ar–H).*;* IR (KBr, υ, cm^−1^): 3086.90(Ar, C–H str), 3567.81 (Ali, C–H str), 1525.42 (Ar, C=C str), 1623.74(C=N), 1327.96 (C–N), 1219.90 (C–O–C Asymmetric) 1038.23 (Ar. C–Cl).;EIMS; Found, (*m/z*): 416.2[M + H]^+^. **C**_27_H_24_N_2_O. Calculated, 416.1*.*

### Pharmacological assay

#### Used animals

Regarding the purpose of investigating acute toxicity, Swiss albino mice (20–25 g) in adulthood along with albino rats (150–200 g) of both genders were used. Six animals of apiece batch were kept separately in polypropylene aluminium cages with rice husk feeding. Mice had been kept at 25–27 °C with an absolute humidity of 30–70%. Before beginning the investigation, the research's strategy had been sanctioned by the institution's IACE (Reg. No. 346/CPCSEA: Dated 03-10-2022).

### Analgesic activity screening

The hot plate test was conducted as previously mentioned. Where the grouping and other conditions were nearly the same as those mentioned above. The hot plate had a metallic surface with dimensions of 20 mm in diameter and 2.0 mm in height. It was set at a temp of 55 °C. Individually mouse was momentarily placed onto a hotplate that was protected from heat loss by a glass beaker. Each mouse also functioned as its control. It was timed how long it took an animal to jump or lick its forepaws. The time it takes for each mouse to respond after licking its forepaws or jumping is known as the latency. The latency in animals not receiving treatment ranged from 5 to 20 s. All groups' latency/reaction times were noted 30 min after injection. Typically, 3 mice were utilised for each group. Each group's mean value was computed and contrasted with the control. To compare the analgesic activity of various medications, diclofenac sodium was utilised as the reference. The following calculation was used to determine the percentage of protection:

$$\left( {{1} - {\text{Vc}}/{\text{Vt}}} \right) \, \times {1}00$$

where Vc is the average number of writhing control animals, and Vt is the average number of writhing test animals.

Turkey–Krammer multiple comparison test was used to analyze the statistical importance, and a p-value of less than 0.05 was regarded as significant.

### Anti-inflammatory activity screening

Using the carrageenan-induced rat hind paw oedema method, the soothing properties of all the produced compounds have been evaluated. Injecting 0.1 mL of a one percent carrageenan solution toward the right hind paw sub-planter region caused the oedema hind paw. Plethysmographic measurements of the paw's thickness were taken both instantly and 180 m after the irritant injection. The volume distinction determined how much oedema occurred. The percentage of oedema inhibition in the three groups—the control, the compound-treated group, and the group receiving the standard drug at 50 mg/kg b.w.—was identified and assessed.

### Statistical analysis

Data in studies involving analgesics as well as anti-inflammatory drugs are reported as means ± SE. Turkey-Kramer multiple comparison testing was used after one-way ANOVA to determine differentiation among the vehicle control also treatment groups. As of that time, statistical importance was defined as a value of probability less than 0.05 (Supplementary Information).

## Supplementary Information

Supplementary Information.

## Data Availability

All required data will be available with the corresponding author upon request.
