# Author Correction: Design, synthesis, and biological evaluation of novel imidazole derivatives as analgesic and anti-inflammatory agents: experimental and molecular docking insights

**DOI:** 10.1038/s41598-024-76905-w

**Published:** 2024-10-25

**Authors:** Gulam Muheyuddeen, Mohd Yaqub Khan, Tanzeem Ahmad, Shriyansh Srivastava, Stuti Verma, Mo. Suheb Ansari, Nilanchala Sahu

**Affiliations:** 1 Department of Pharmaceutical Chemistry, Faculty of Pharmacy, Jahangirabad Institute of Technology, Jahangirabad Fort, Jahangirabad, Barabanki, 225203 Uttar Pradesh India; 2 Department of Biomedical Engineering, Chung Yuan Christian University, Taoyuan, Taiwan; 3 Department of Pharmacy, Integral University, Lucknow, Uttar Pradesh India; 4 Department of Pharmacy, School of Medical and Allied Sciences, Galgotias University, Greater Noida, Uttar Pradesh India; 5 Department of Pharmacology, Delhi Pharmaceutical Sciences and Research University (DPSRU), Sector 3 Pushp Vihar, New Delhi, 110017 India; 6 Department of Pharmacy, Aryakul College of Pharmacy and Research, Sitapur Village, Jajjaur, Post, Manawa (Near Krishi Vigyan Kendra Sitapur) Sidhauli, Sitapur, Uttar Pradesh India; 7 Sharda School of Pharmacy, Sharda University, Greater Noida, 201310 Uttar Pradesh India

Correction to: *Scientific Reports*10.1038/s41598-024-72399-8, published online 04 October 2024

In the original version of this Article, Nilanchala Sahu was incorrectly affiliated with ‘Department of Pharmaceutics, Faculty of Pharmacy, Jahangirabad Institute of Technology, Jahangirabad Fort, Jahangirabad, Barabanki 225203, Uttar Pradesh, India’. The correct affiliation is listed below.

Sharda School of Pharmacy, Sharda University, Greater Noida 201310, Uttar Pradesh, India.

The original Article has been corrected.

