# Supplementary material for: Design, synthesis, and biological evaluation of novel imidazole derivatives as analgesic and anti-inflammatory agents: experimental and molecular docking insights
Source: Sci Rep. 2024 Oct 4;14:23121. doi: 10.1038/s41598-024-72399-8 (PMC11452658; doi:10.1038/s41598-024-72399-8)
Supplement: Supplementary file 1 — Supplementary Information. [file 41598_2024_72399_MOESM1_ESM.pdf]

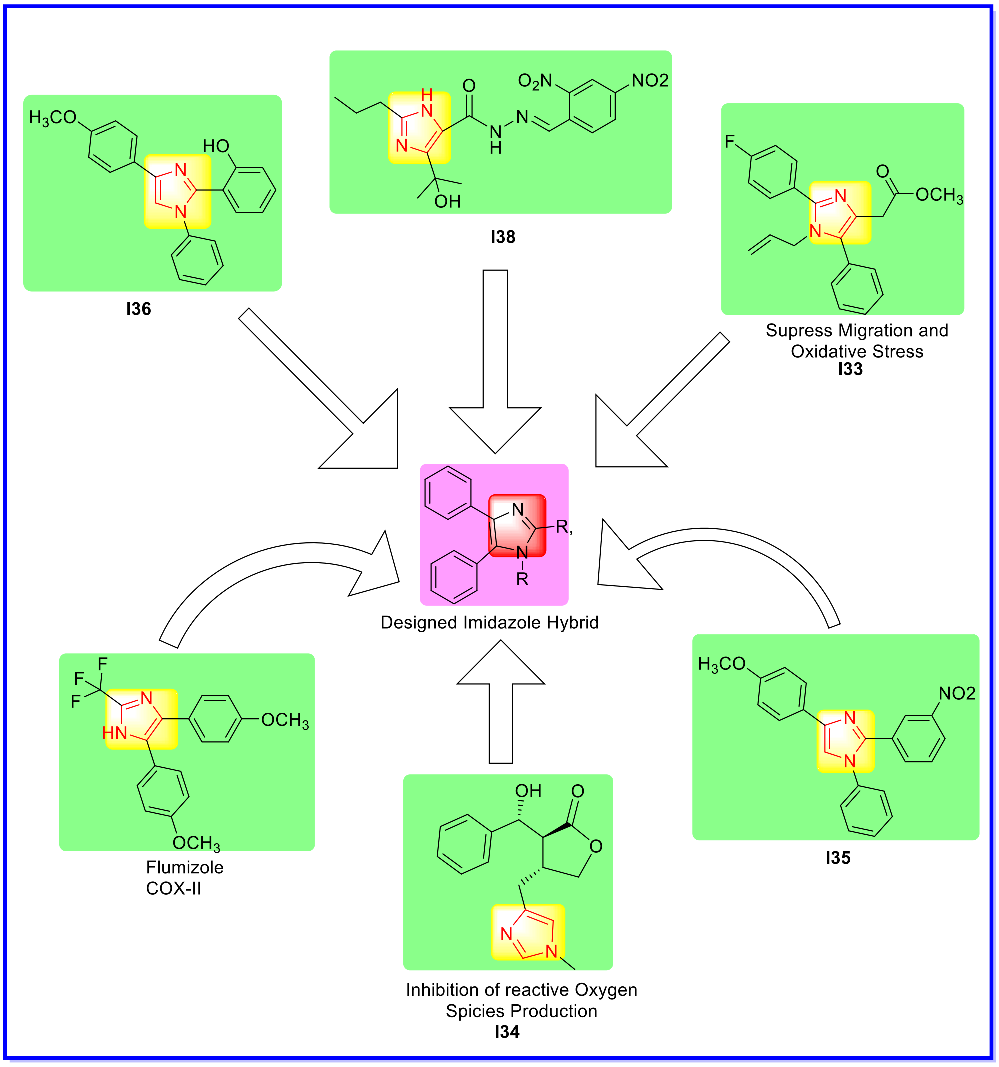

Supplementary Figure 1: Rational drug design approach used to prepare Imidazole hybrids.

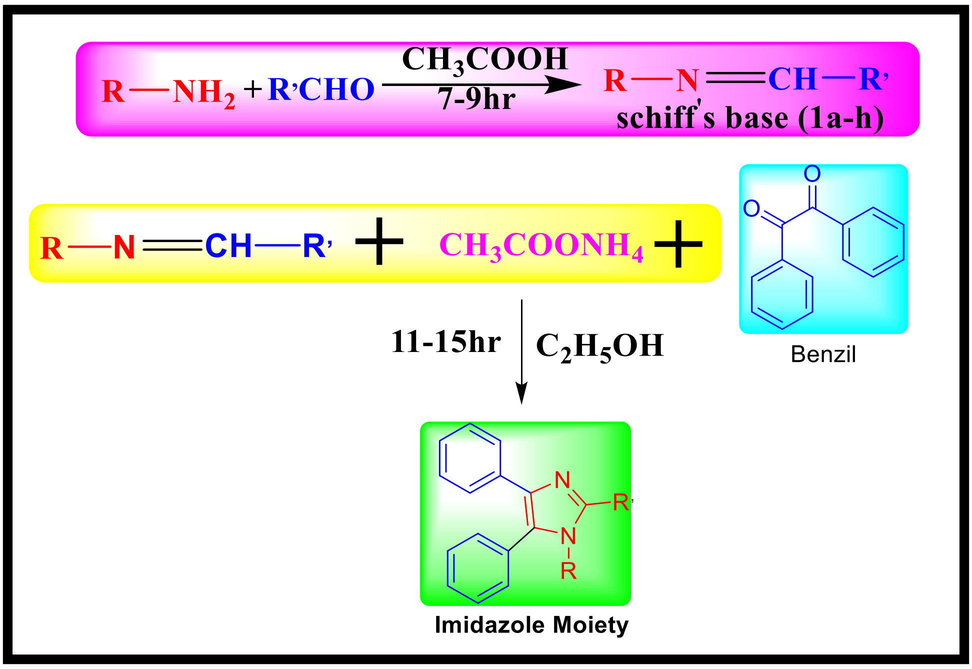

Supplementary Figure 2: Schematic representation of 1, 3-diaza-2, 4-cyclopentadiene derivatives (2a-h)

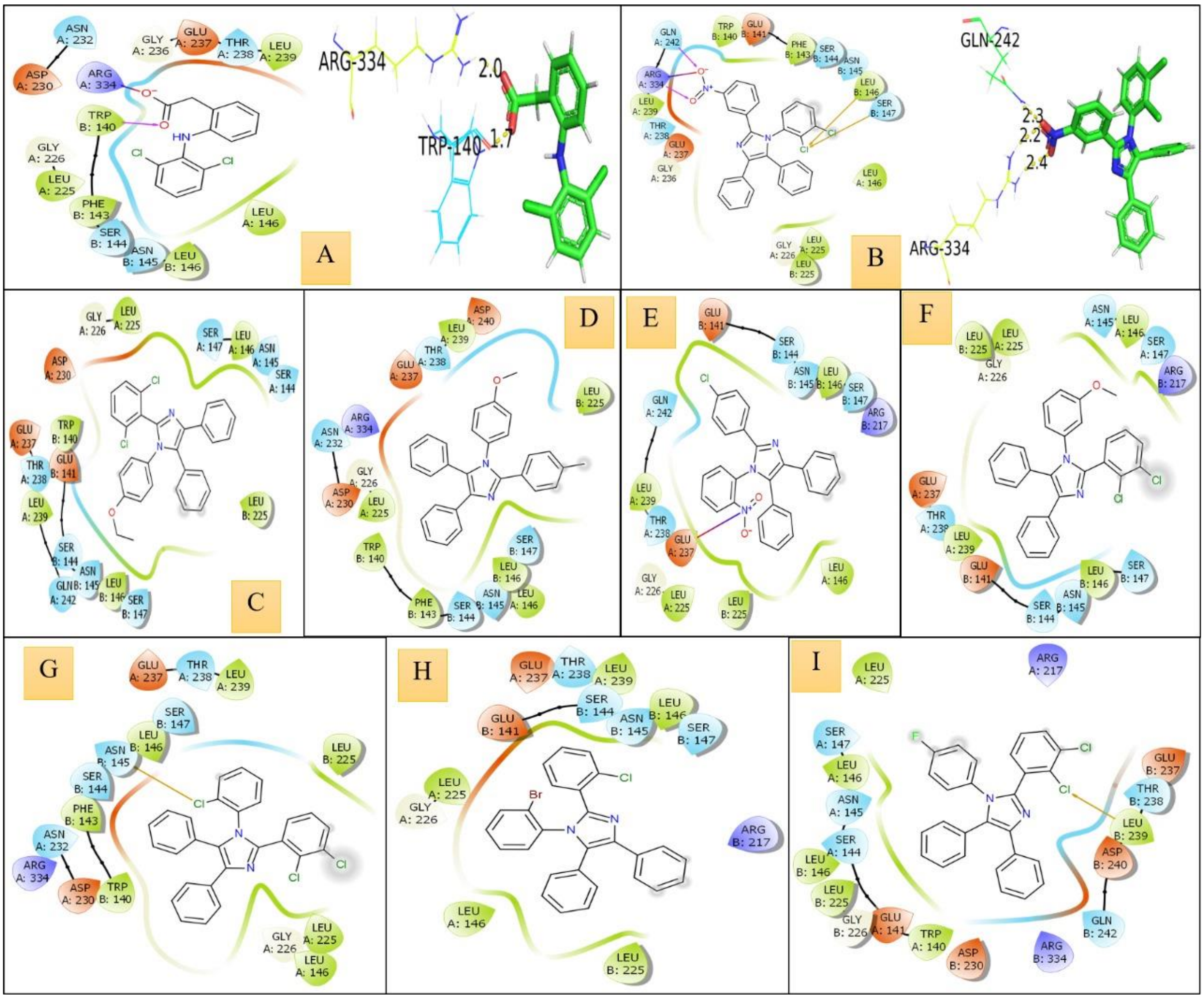

Supplementary Figure 3: Molecular docking analysis results of COX-2 with different compounds obtained from Schrodinger molecular visualizer (2D diagrams) and PyMOL for 3D diagrams. (A: Diclofenac-standard; B: 2g; C: 2a; D: 2h; E: 2c; F: 2e; G: 2b; H: 2f; I: 2d)

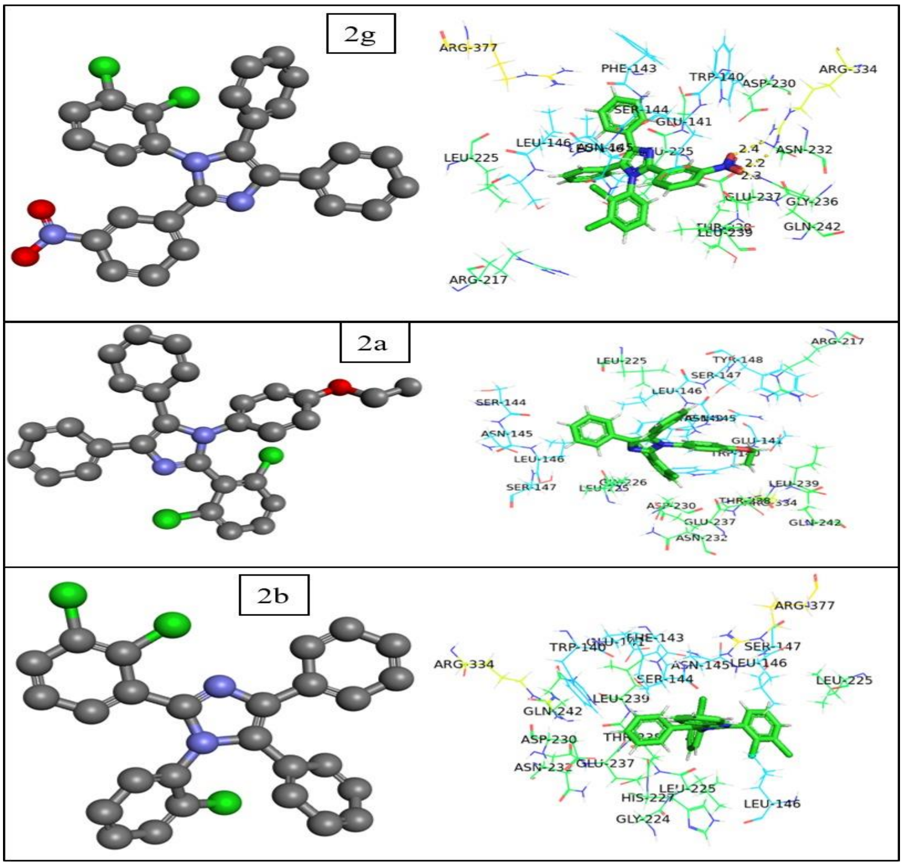

Supplementary Figure 4: Molecular docking analysis results of COX-2 with different compounds obtained PyMOL for 3D diagrams of potent compounds. (B: 2g; C: 2a; G: 2b)

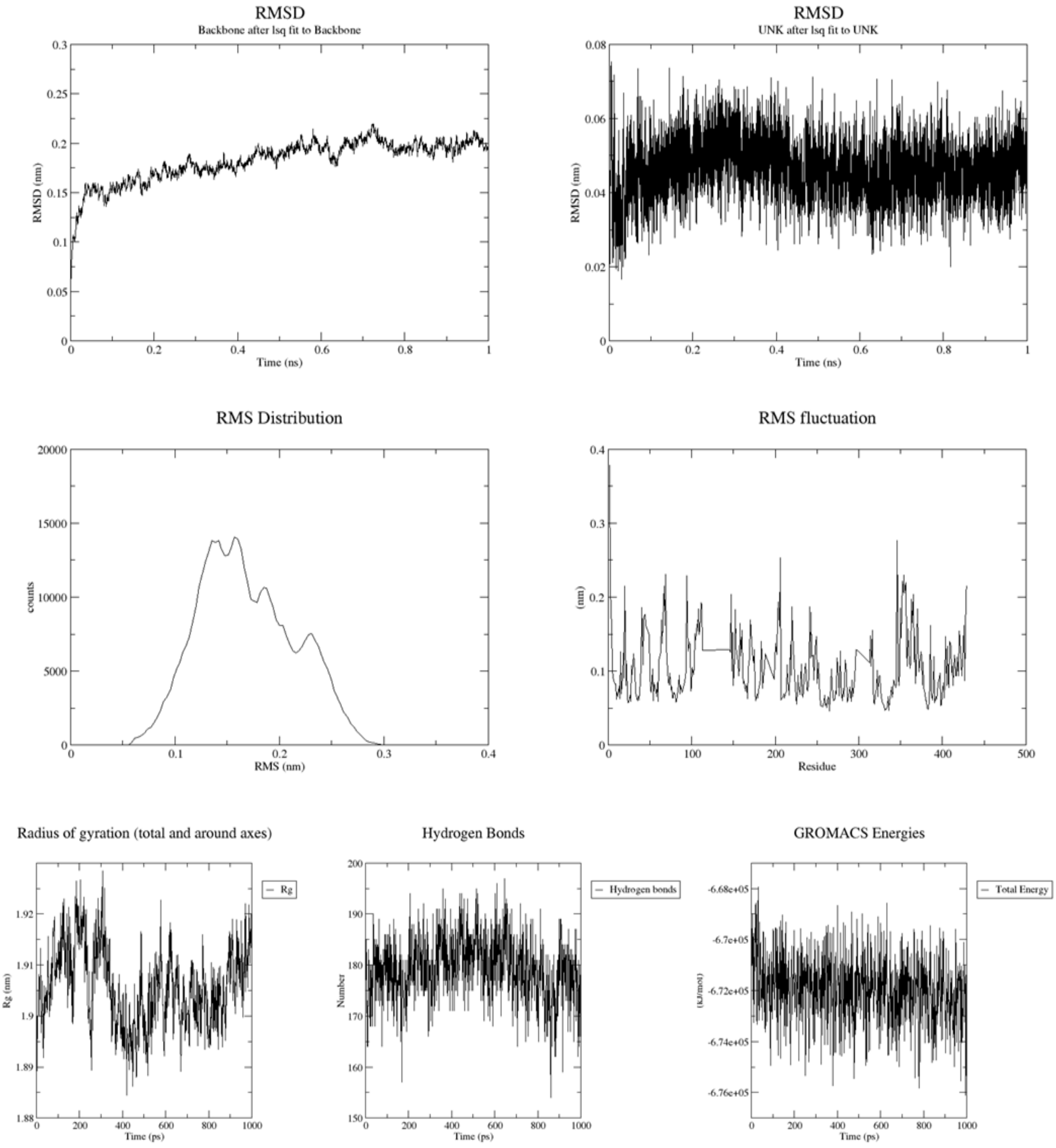

Supplementary Figure 5: (Simulation parameters for 4PH9 and 2g compound-complex)

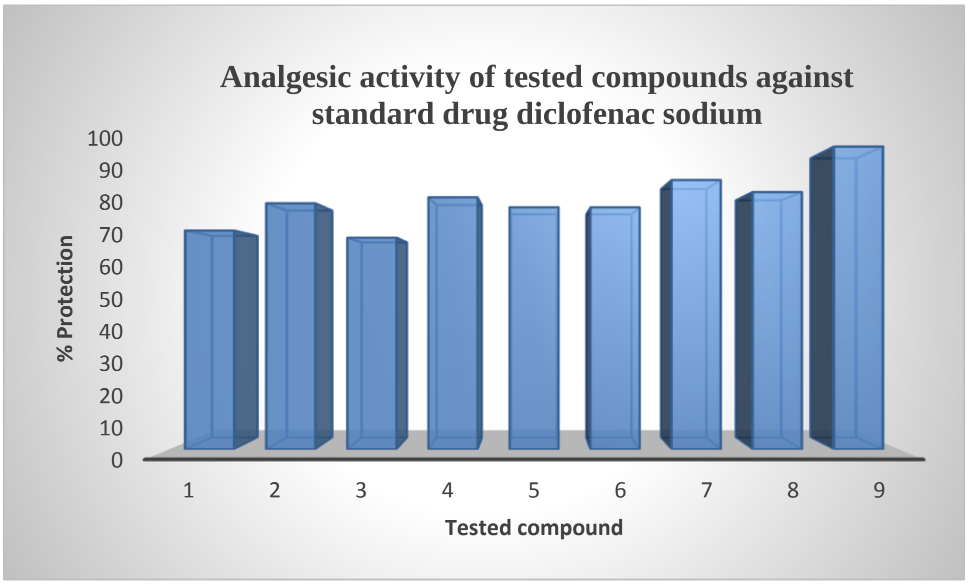

Supplementary Figure 6: **A**nalgesic effect of tested analogues at (100 mg/kg b.w) and Diclofenac sodium (50 mg/kg b.w). Concentration has been analysed

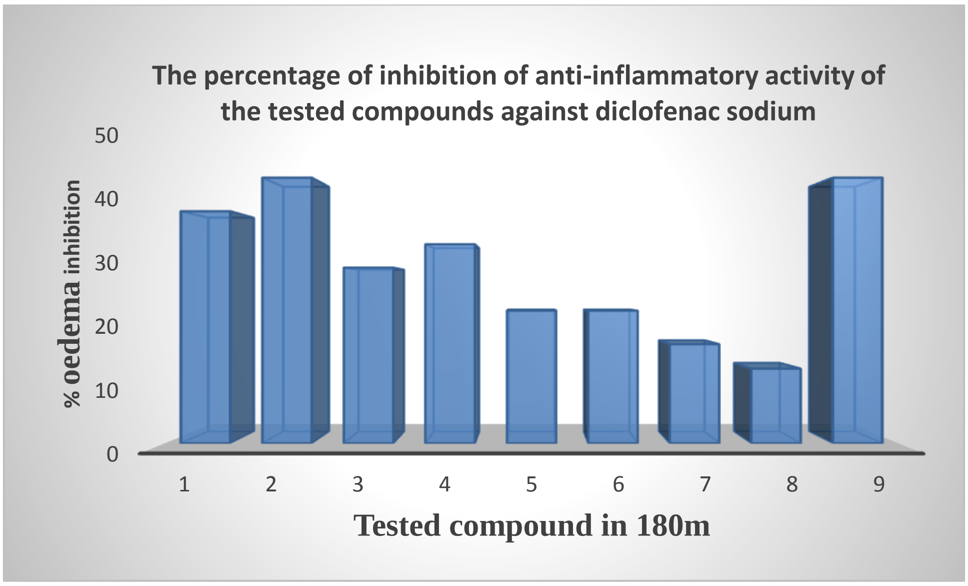

Supplementary Figure 7: % inhibition of the soothing effect of the investigated agents (100 mg/kg, b.w) and diclofenac (50mg/kg, b.w) in 180 m.

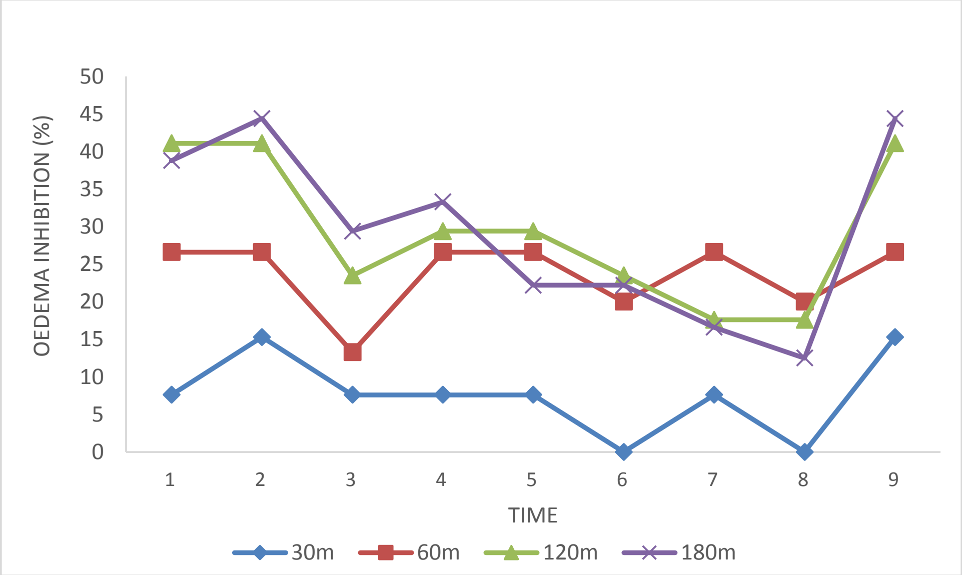

Supplementary Figure 8: The inhibition of the soothing effect of the investigated agents (100 mg/kg, b.w) and diclofenac (50mg/kg, b.w)

NAME 22053184.IKO.1EXPNO 12

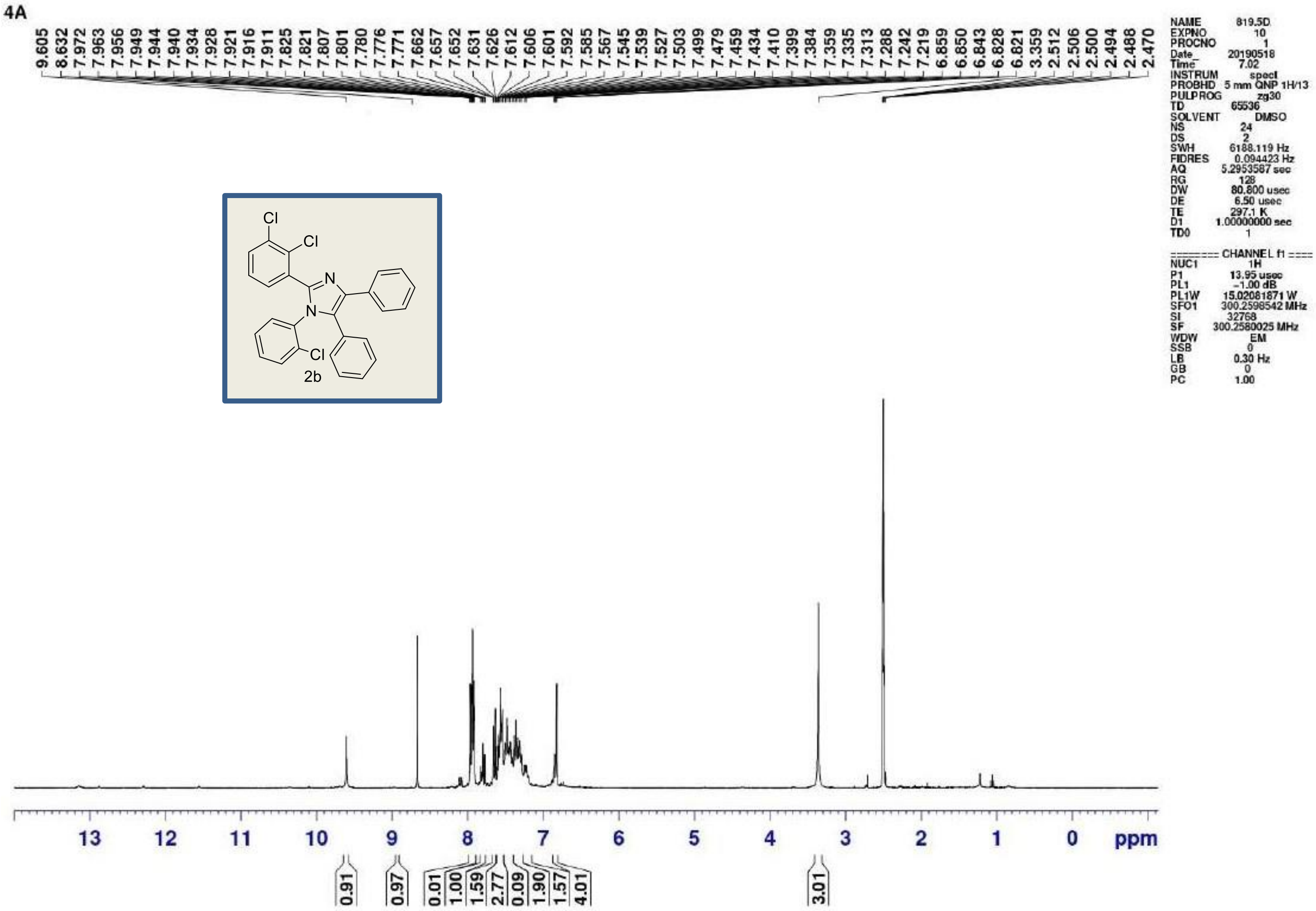

Supplementary Figure 9:

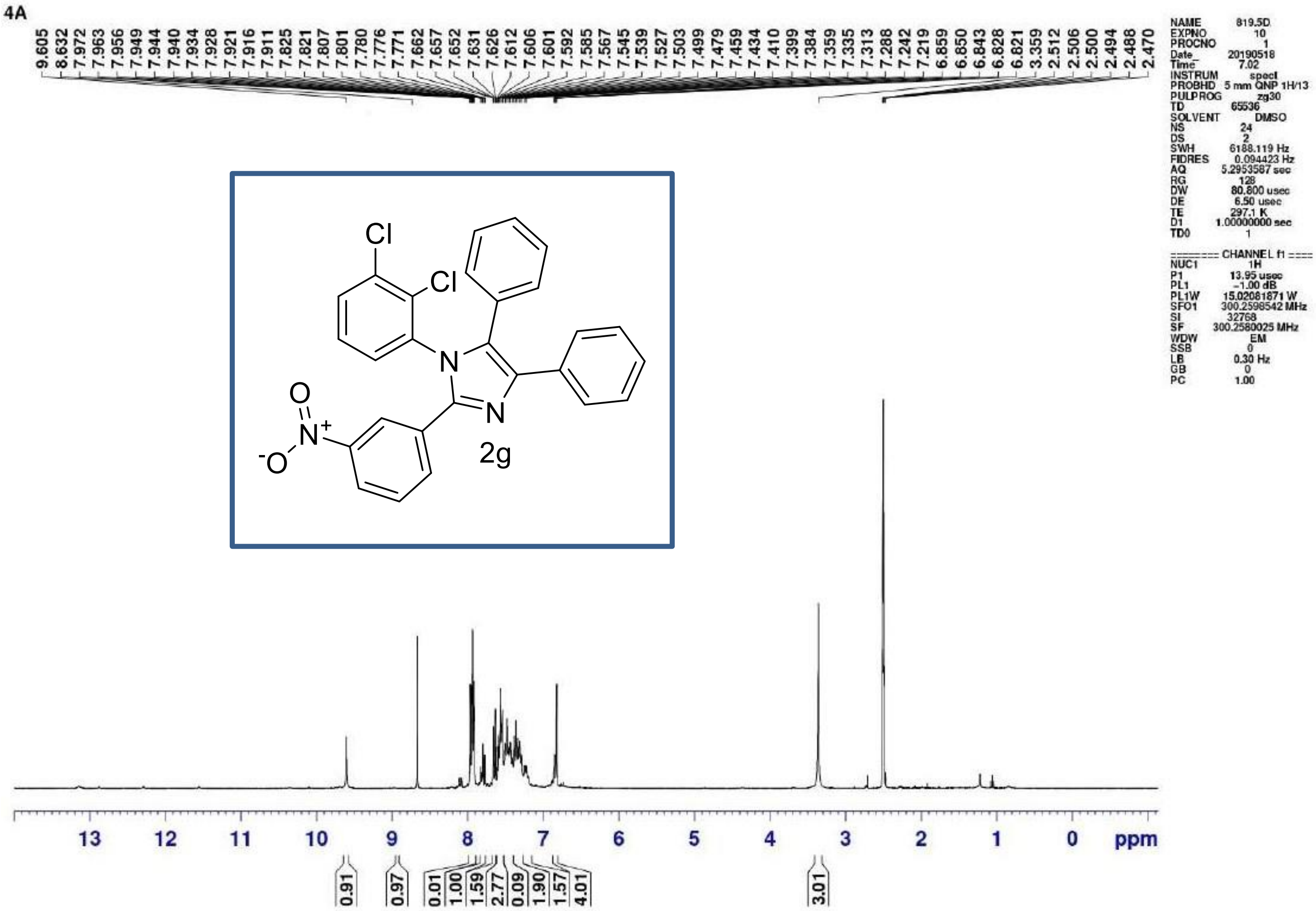

Supplementary Figure 10:

Supplementary Table 1. 2D and 3D diagram of molecular docking results.

<table>
<tr><th>Docking complex with compound</th><th>2D diagram</th><th>3D diagram</th></tr>
<tr><td>2A</td><td>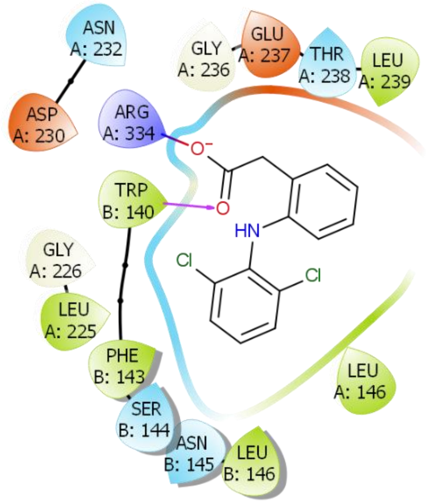
</td><td>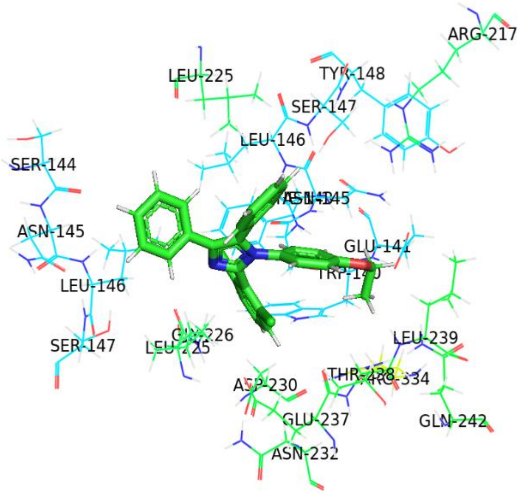
</td></tr>
<tr><td>2B</td><td>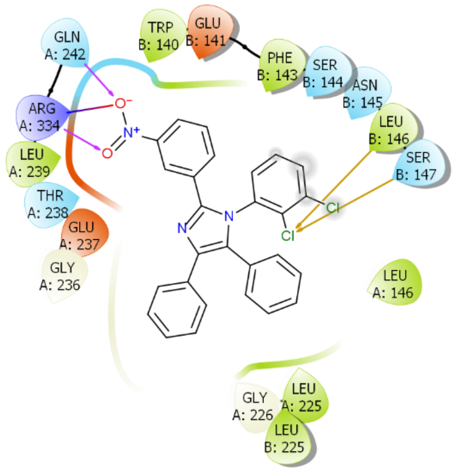
</td><td>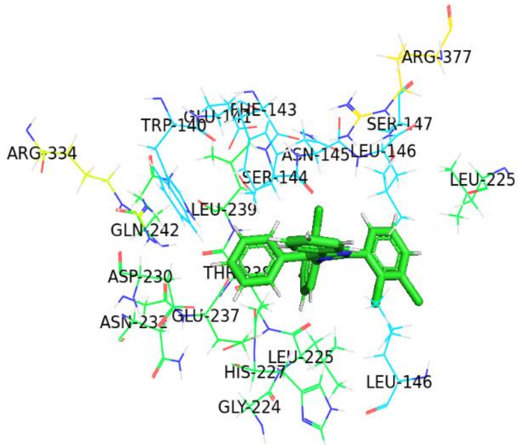
</td></tr>
<tr><td>2C</td><td>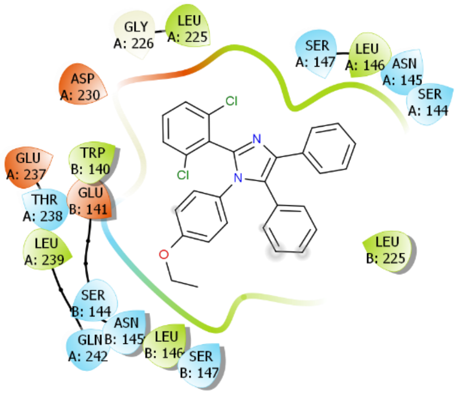
</td><td>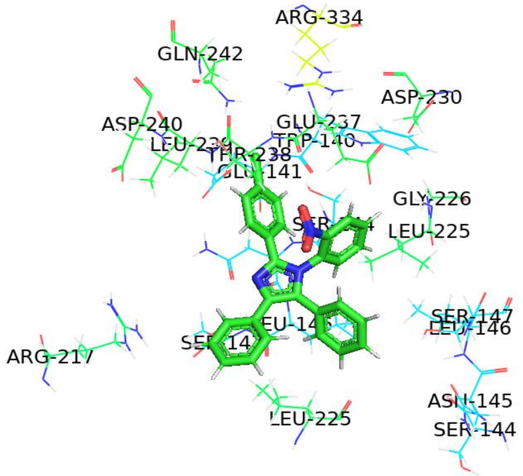
</td></tr>
</table>

2D

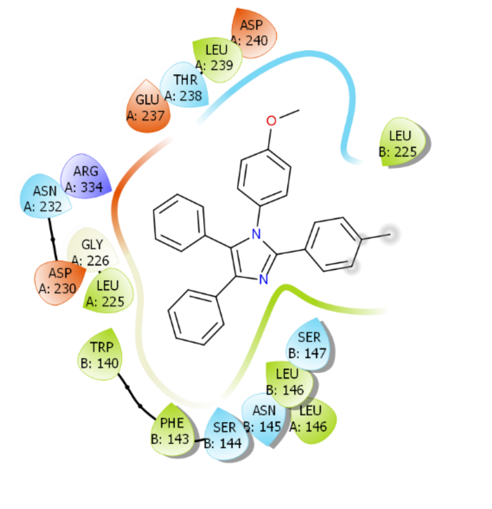
ASP A: 240
LEU A: 239
THR A: 238
GLU A: 237
LEU B: 225
ARG A: 334
ASN A: 232
GLY A: 226
ASP A: 230
LEU A: 225
TRP B: 140
SER B: 147
LEU B: 146
ASN B: 145
LEU A: 146
PHE B: 143
SER B: 144
O
N
N

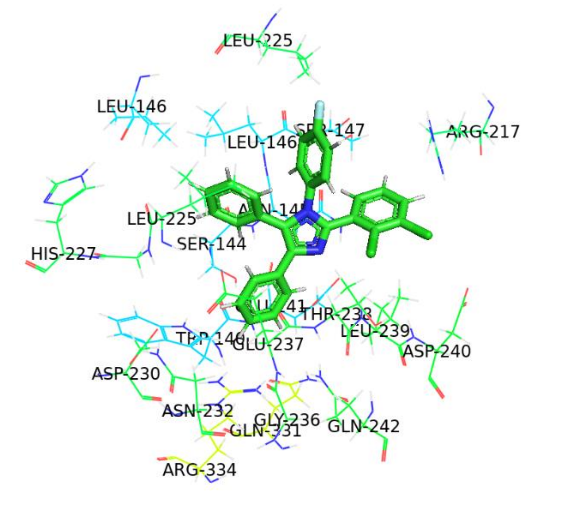
LEU-225
LEU-146
LEU-146
SER-147
ARG-217
LEU-225
SER-144
HIS-227
THR-232
LEU-239
ASP-240
GLU-237
ASP-230
ASN-232
GLY-236
GLN-331
GLN-242
ARG-334

2E

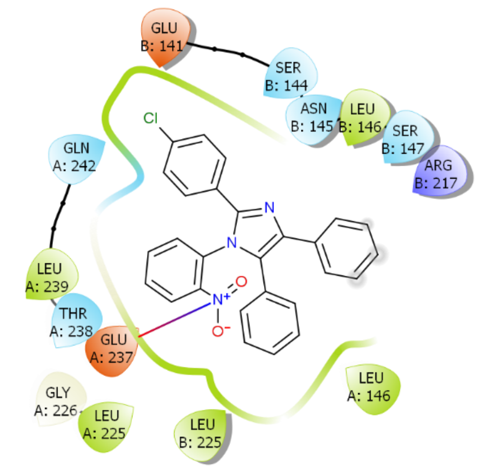
GLU B: 141
SER B: 144
ASN B: 145
LEU B: 146
SER B: 147
ARG B: 217
GLN A: 242
LEU A: 239
THR A: 238
GLU A: 237
GLY A: 226
LEU A: 225
LEU B: 225
LEU A: 146
Cl
N
N
N^{+}
O
O^{-}

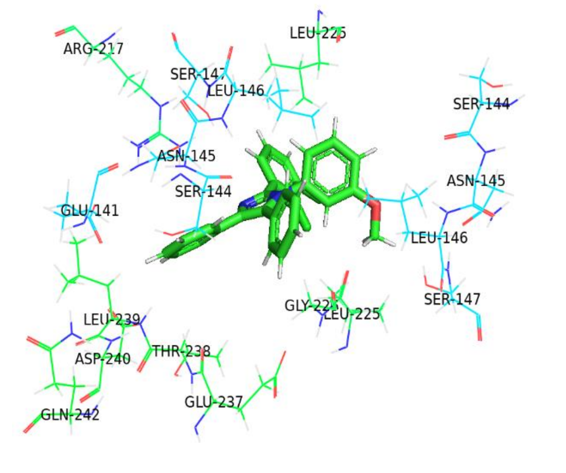
ARG-217
LEU-225
SER-147
LEU-146
SER-144
ASN-145
SER-144
ASN-145
GLU-141
LEU-146
SER-147
LEU-239
ASP-240
THR-238
LEU-225
GLU-237
GLN-242

2F

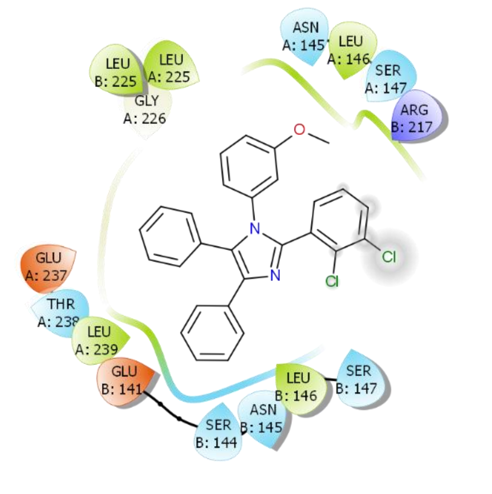
ASN A: 145
LEU A: 146
SER A: 147
ARG B: 217
LEU B: 225
LEU A: 225
GLY A: 226
GLU A: 237
THR A: 238
LEU A: 239
GLU B: 141
LEU B: 146
SER B: 147
ASN B: 145
SER B: 144
O
N
N
Cl
Cl

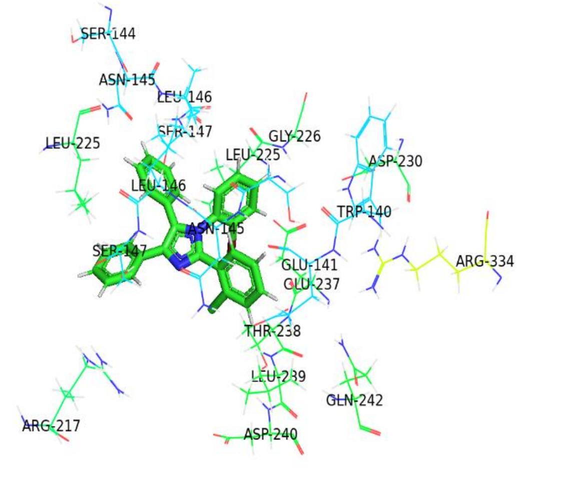
SER-144
ASN-145
LEU-146
SER-147
LEU-225
GLY-226
LEU-225
ASP-230
LEU-146
TRP-140
ASN-145
SER-147
GLU-141
GLU-237
ARG-334
THR-238
LEU-239
GLN-242
ARG-217
ASP-240

2G

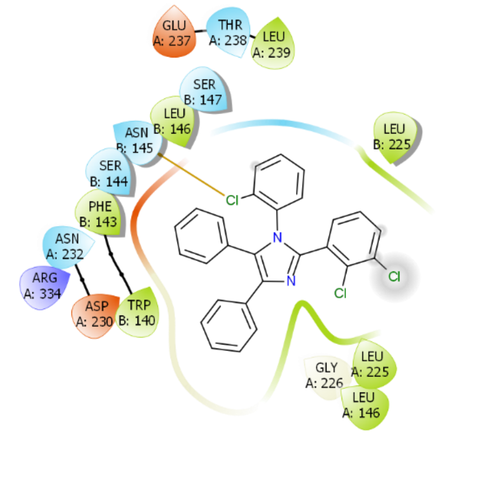
GLU A: 237
THR A: 238
LEU A: 239
SER B: 147
LEU B: 146
ASN B: 145
LEU B: 225
SER B: 144
PHE B: 143
ASN A: 232
ARG A: 334
ASP A: 230
TRP B: 140
GLY A: 226
LEU A: 225
LEU A: 146
Cl
N
N
Cl
Cl

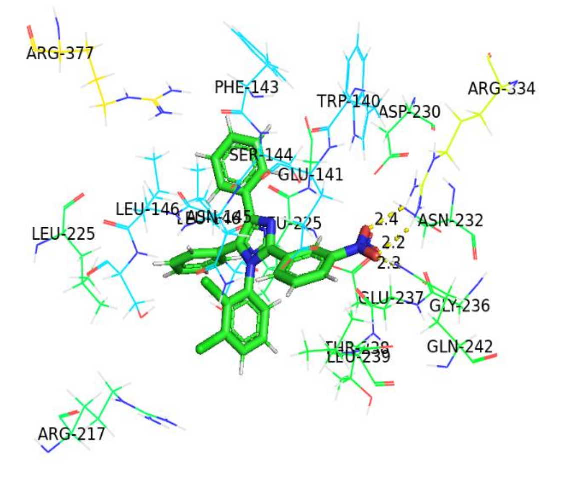
ARG-377
PHE-143
TRP-140
ASP-230
ARG-334
SER-144
GLU-141
LEU-146
ASN-145
ASN-232
LEU-225
2.4
2.2
2.3
GLU-237
GLY-236
GLN-242
ARG-217

2H	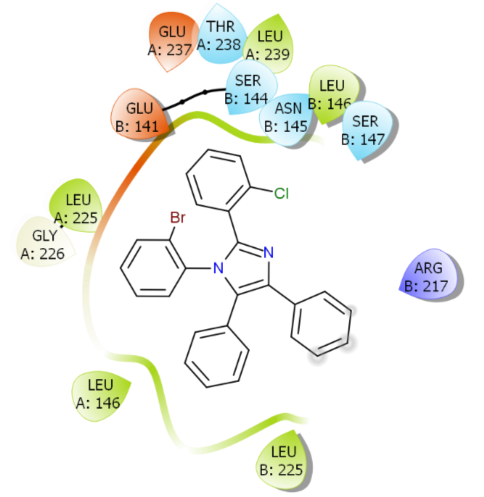	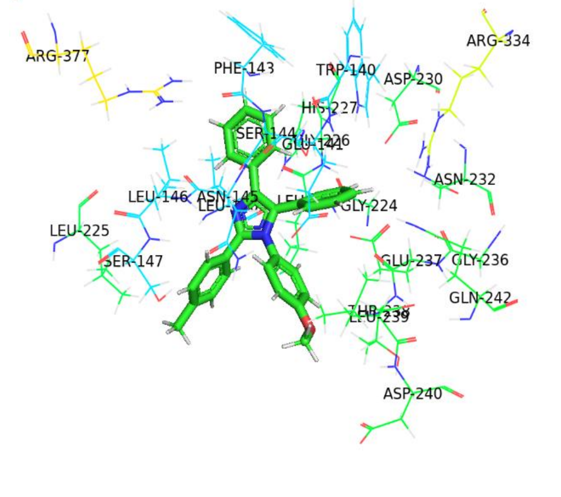

Supplementary Table 2. 2D and 3D diagram of potentially active compounds.

**Compound id**	**3D structure**	**2D Structure**
2A	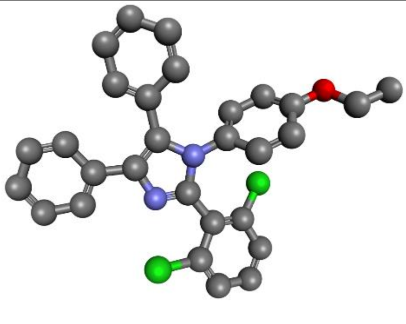	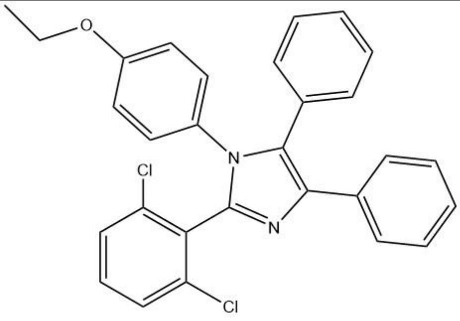

2B	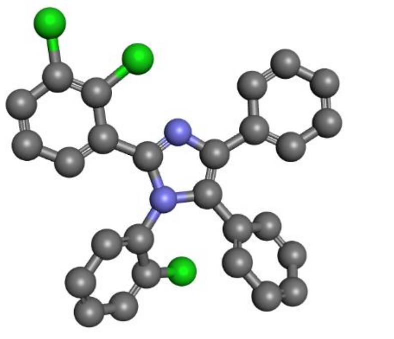	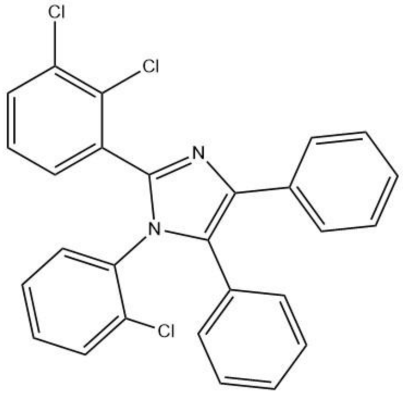Cl<br>Cl<br>N<br>N<br>Cl
2C	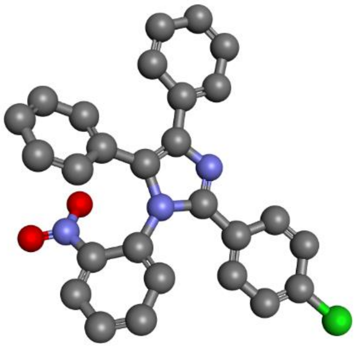	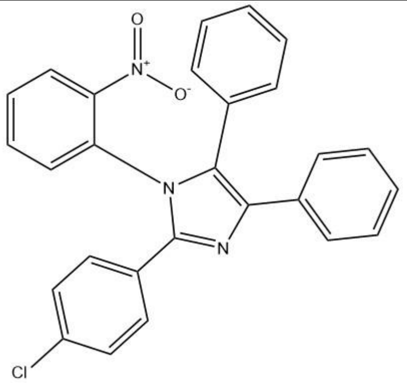O<br>N+<br>O-<br>N<br>N<br>Cl
2D	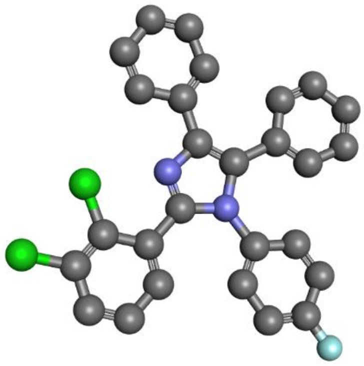	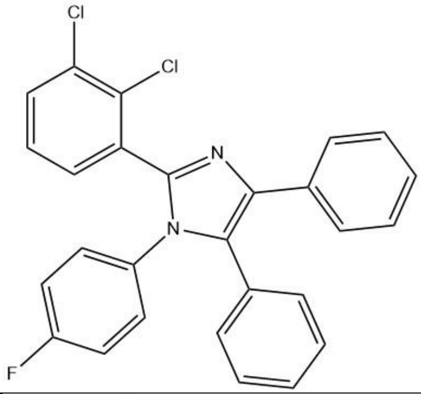Cl<br>Cl<br>N<br>N<br>F
2E	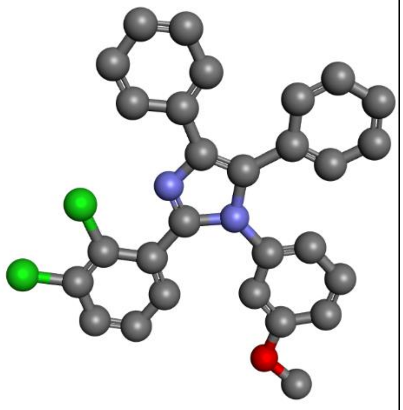	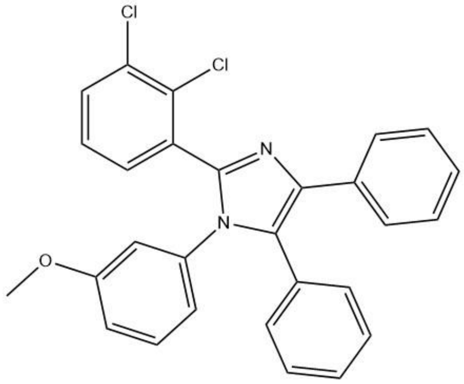Cl<br>Cl<br>N<br>O<br>N

2F	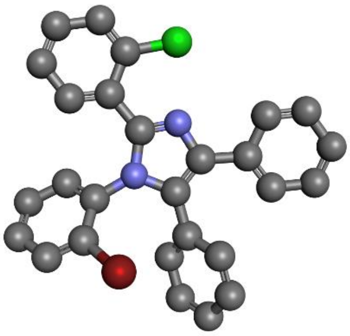	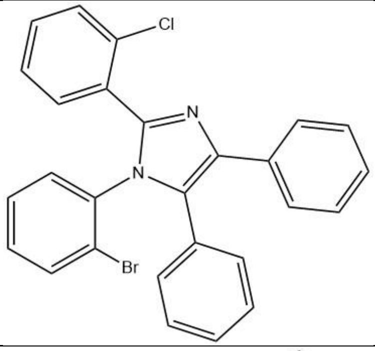
2g	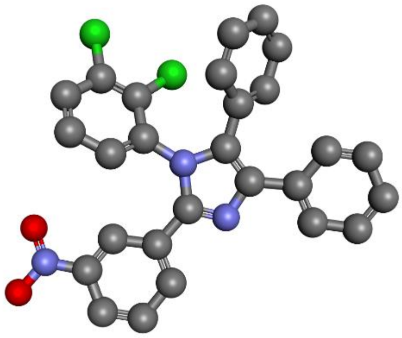	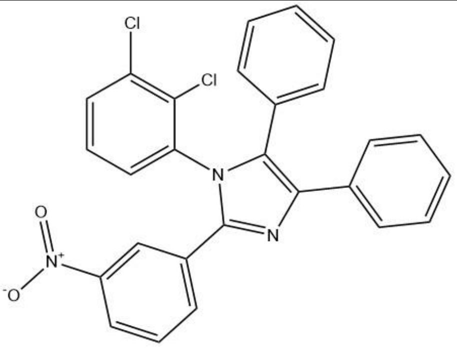
2H	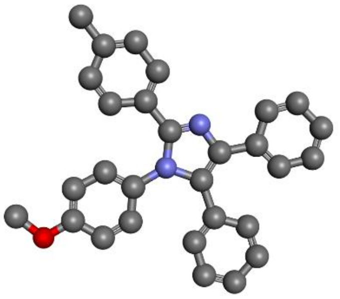	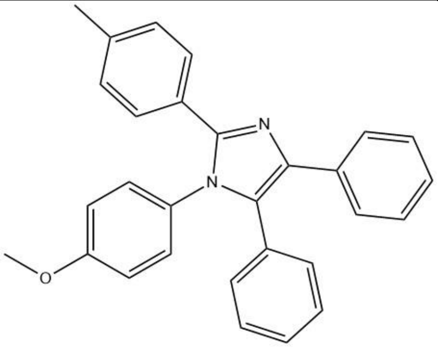

Oral toxicity prediction results for input compound

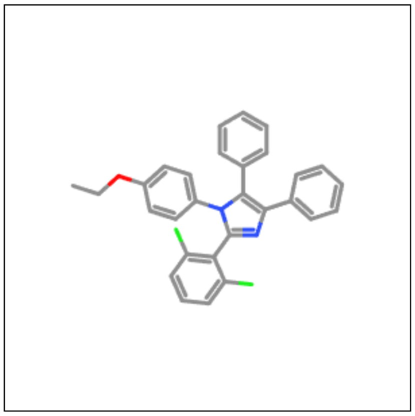

Predicted LD50: 2000mg/kg

Predicted Toxicity Class: 4

1	2	3	4	5	6

Average similarity: 55.3%

Prediction accuracy: 67.38%

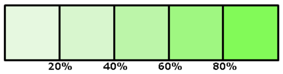

Name	
Molweight	485.4
Number of hydrogen bond acceptors	2
Number of hydrogen bond donors	0
Number of atoms	34
Number of bonds	38
Number of rotable bonds	6
Molecular refractivity	141.19
Topological Polar Surface Area	27.05
octanol/water partition coefficient(logP)	8.58

Toxicity Model Report

Copy Excel CSV PDF

Classification	Target	Shorthand	Prediction	Probability
Organ toxicity	Hepatotoxicity	dili	Inactive	0.58
Organ toxicity	Neurotoxicity	neuro	**Active**	0.83
Organ toxicity	Nephrotoxicity	nephro	Inactive	0.61
Organ toxicity	Respiratory toxicity	respi	**Active**	0.65
Organ toxicity	Cardiotoxicity	cardio	Inactive	0.78
Toxicity end points	Carcinogenicity	carcino	**Active**	0.52
Toxicity end points	Immunotoxicity	immuno	Inactive	0.91
Toxicity end points	Mutagenicity	mutagen	Inactive	0.60
Toxicity end points	Cytotoxicity	cyto	Inactive	0.63
Toxicity end points	BBB-barrier	bbb	**Active**	0.85
Toxicity end points	Ecotoxicity	eco	**Active**	0.75
Toxicity end points	Clinical toxicity	clinical	**Active**	0.63
Toxicity end points	Nutritional toxicity	nutri	Inactive	0.70
Tox21-Nuclear receptor signalling pathways	Aryl hydrocarbon Receptor (AhR)	nr_ahr	Inactive	0.58
Tox21-Nuclear receptor signalling pathways	Androgen Receptor (AR)	nr_ar	Inactive	0.96
Tox21-Nuclear receptor signalling pathways	Androgen Receptor Ligand Binding Domain (AR-LBD)	nr_ar_lbd	Inactive	0.99
Tox21-Nuclear receptor signalling pathways	Aromatase	nr_aromatase	**Active**	0.56
Tox21-Nuclear receptor signalling pathways	Estrogen Receptor Alpha (ER)	nr_er	Inactive	0.81
Tox21-Nuclear receptor signalling pathways	Estrogen Receptor Ligand Binding Domain (ER-LBD)	nr_er_lbd	Inactive	0.96
Tox21-Nuclear receptor signalling pathways	Peroxisome Proliferator Activated Receptor Gamma (PPAR-Gamma)	nr_ppar_gamma	Inactive	0.87
Tox21-Stress response pathways	Nuclear factor (erythroid-derived 2)-like 2/antioxidant responsive element (nrf2/ARE)	sr_are	Inactive	0.66
Tox21-Stress response pathways	Heat shock factor response element (HSE)	sr_hse	Inactive	0.66
Tox21-Stress response pathways	Mitochondrial Membrane Potential (MMP)	sr_mmp	Inactive	0.67
Tox21-Stress response pathways	Phosphoprotein (Tumor Supressor) p53	sr_p53	Inactive	0.79
Tox21-Stress response pathways	ATPase family AAA domain-containing protein 5 (ATAD5)	sr_atad5	Inactive	0.87
Molecular Initiating Events	Thyroid hormone receptor alpha (THRα)	mie_thr_alpha	Inactive	0.90
Molecular Initiating Events	Thyroid hormone receptor beta (THRβ)	mie_thr_beta	Inactive	0.78
Molecular Initiating Events	Transtyretrin (TTR)	mie_ttr	Inactive	0.97
Molecular Initiating Events	Ryanodine receptor (RYR)	mie_ryr	Inactive	0.98
Molecular Initiating Events	GABA receptor (GABAR)	mie_gabar	Inactive	0.96
Molecular Initiating Events	Glutamate N-methyl-D-aspartate receptor (NMDAR)	mie_nmdar	Inactive	0.92
Molecular Initiating Events	alpha-amino-3-hydroxy-5-methyl-4-isoxazolepropionate receptor (AMPAR)	mie_ampar	Inactive	0.97

Classification	Target	Shorthand	Prediction	Probability
Molecular Initiating Events	Kainate receptor (KAR)	mie_kar	Inactive	0.99
Molecular Initiating Events	Achetylcholinesterase (AChE)	mie_ache	Inactive	0.58
Molecular Initiating Events	Constitutive androstane receptor (CAR)	mie_car	Inactive	0.98
Molecular Initiating Events	Pregnane X receptor (PXR)	mie_pxr	Inactive	0.92
Molecular Initiating Events	NADH-quinone oxidoreductase (NADHOX)	mie_nadhox	Inactive	0.97
Molecular Initiating Events	Voltage gated sodium channel (VGSC)	mie_vgsc	Inactive	0.95
Molecular Initiating Events	Na+/I- symporter (NIS)	mie_nis	Inactive	0.98
Metabolism	Cytochrome CYP1A2	CYP1A2	**Active**	0.64
Metabolism	Cytochrome CYP2C19	CYP2C19	**Active**	0.73
Metabolism	Cytochrome CYP2C9	CYP2C9	Inactive	0.50
Metabolism	Cytochrome CYP2D6	CYP2D6	**Active**	0.74
Metabolism	Cytochrome CYP3A4	CYP3A4	**Active**	0.61
Metabolism	Cytochrome CYP2E1	CYP2E1	Inactive	0.99

Toxicity targets

Possible binding to toxicity targets is shown below. For more information on the targets, please click on the individual abbreviations.

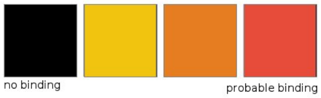

AA2AR	ADRB2	ANDR	AOFA	CRFR1	DRD3	ESR1	ESR2	GCR	HRH1	NR1I2	OPRK	OPRM	PDE4D	PGH1	PRGR

Details about possible toxicity targets:

	Toxicity Target	Avg Pharmacophore Fit	Avg Similarity Known Ligands
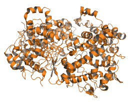	Prostaglandin G/H Synthase 1	0%	79.59%

Oral toxicity prediction results for input compound

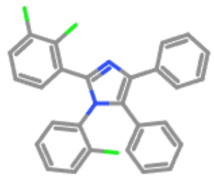

Predicted LD50: 938mg/kg

Predicted Toxicity Class: 4

1	2	3	4	5	6

Average similarity: 48.03%

Prediction accuracy: 54.26%

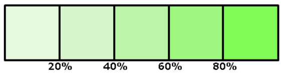

Name	
Molweight	475.8
Number of hydrogen bond acceptors	1
Number of hydrogen bond donors	0
Number of atoms	32
Number of bonds	36
Number of rotable bonds	4
Molecular refractivity	134.9
Topological Polar Surface Area	17.82
octanol/water partition coefficient(logP)	8.83

Toxicity Model Report

Copy Excel CSV PDF

Classification	Target	Shorthand	Prediction	Probability
Organ toxicity	Hepatotoxicity	dili	Inactive	0.52
Organ toxicity	Neurotoxicity	neuro	**Active**	0.89
Organ toxicity	Nephrotoxicity	nephro	Inactive	0.86
Organ toxicity	Respiratory toxicity	respi	**Active**	0.57
Organ toxicity	Cardiotoxicity	cardio	Inactive	0.92
Toxicity end points	Carcinogenicity	carcino	**Active**	0.53
Toxicity end points	Immunotoxicity	immuno	Inactive	0.94
Toxicity end points	Mutagenicity	mutagen	Inactive	0.61
Toxicity end points	Cytotoxicity	cyto	Inactive	0.88
Toxicity end points	BBB-barrier	bbb	**Active**	0.91
Toxicity end points	Ecotoxicity	eco	**Active**	0.75
Toxicity end points	Clinical toxicity	clinical	**Active**	0.62
Toxicity end points	Nutritional toxicity	nutri	Inactive	0.68
Tox21-Nuclear receptor signalling pathways	Aryl hydrocarbon Receptor (AhR)	nr_ahr	Inactive	0.51
Tox21-Nuclear receptor signalling pathways	Androgen Receptor (AR)	nr_ar	Inactive	0.97
Tox21-Nuclear receptor signalling pathways	Androgen Receptor Ligand Binding Domain (AR-LBD)	nr_ar_lbd	Inactive	0.99
Tox21-Nuclear receptor signalling pathways	Aromatase	nr_aromatase	**Active**	0.68
Tox21-Nuclear receptor signalling pathways	Estrogen Receptor Alpha (ER)	nr_er	Inactive	0.78
Tox21-Nuclear receptor signalling pathways	Estrogen Receptor Ligand Binding Domain (ER-LBD)	nr_er_lbd	Inactive	0.88
Tox21-Nuclear receptor signalling pathways	Peroxisome Proliferator Activated Receptor Gamma (PPAR-Gamma)	nr_ppar_gamma	Inactive	0.97
Tox21-Stress response pathways	Nuclear factor (erythroid-derived 2)-like 2/antioxidant responsive element (nrf2/ARE)	sr_are	**Active**	0.50
Tox21-Stress response pathways	Heat shock factor response element (HSE)	sr_hse	**Active**	0.50
Tox21-Stress response pathways	Mitochondrial Membrane Potential (MMP)	sr_mmp	Inactive	0.70
Tox21-Stress response pathways	Phosphoprotein (Tumor Supressor) p53	sr_p53	Inactive	0.79
Tox21-Stress response pathways	ATPase family AAA domain-containing protein 5 (ATAD5)	sr_atad5	Inactive	0.81
Molecular Initiating Events	Thyroid hormone receptor alpha (THRα)	mie_thr_alpha	Inactive	0.90
Molecular Initiating Events	Thyroid hormone receptor beta (THRβ)	mie_thr_beta	Inactive	0.78
Molecular Initiating Events	Transtyretrin (TTR)	mie_ttr	Inactive	0.97
Molecular Initiating Events	Ryanodine receptor (RYR)	mie_ryr	Inactive	0.98
Molecular Initiating Events	GABA receptor (GABAR)	mie_gabar	Inactive	0.96
Molecular Initiating Events	Glutamate N-methyl-D-aspartate receptor (NMDAR)	mie_nmdar	Inactive	0.92
Molecular Initiating Events	alpha-amino-3-hydroxy-5-methyl-4-isoxazolepropionate receptor (AMPAR)	mie_ampar	Inactive	0.97

Classification	Target	Shorthand	Prediction	Probability
Molecular Initiating Events	Kainate receptor (KAR)	mie_kar	Inactive	0.99
Molecular Initiating Events	Achetylcholinesterase (AChE)	mie_ache	Inactive	0.52
Molecular Initiating Events	Constitutive androstane receptor (CAR)	mie_car	Inactive	0.98
Molecular Initiating Events	Pregnane X receptor (PXR)	mie_pxr	Inactive	0.92
Molecular Initiating Events	NADH-quinone oxidoreductase (NADHOX)	mie_nadhox	Inactive	0.97
Molecular Initiating Events	Voltage gated sodium channel (VGSC)	mie_vgsc	Inactive	0.95
Molecular Initiating Events	Na+/I- symporter (NIS)	mie_nis	Inactive	0.98
Metabolism	Cytochrome CYP1A2	CYP1A2	**Active**	0.82
Metabolism	Cytochrome CYP2C19	CYP2C19	**Active**	0.94
Metabolism	Cytochrome CYP2C9	CYP2C9	**Active**	0.52
Metabolism	Cytochrome CYP2D6	CYP2D6	**Active**	0.83
Metabolism	Cytochrome CYP3A4	CYP3A4	**Active**	0.91
Metabolism	Cytochrome CYP2E1	CYP2E1	Inactive	0.96

Toxicity targets

Possible binding to toxicity targets is shown below. For more information on the targets, please click on the individual abbreviations.

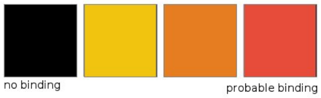

AA2AR	ADRB2	ANDR	AOFA	CRFR1	DRD3	ESR1	ESR2	GCR	HRH1	NR1I2	OPRK	OPRM	PDE4D	PGH1	PRGR

Details about possible toxicity targets:

	Toxicity Target	Avg Pharmacophore Fit	Avg Similarity Known Ligands
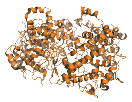	Prostaglandin G/H Synthase 1	0%	74.32%

Oral toxicity prediction results for input compound

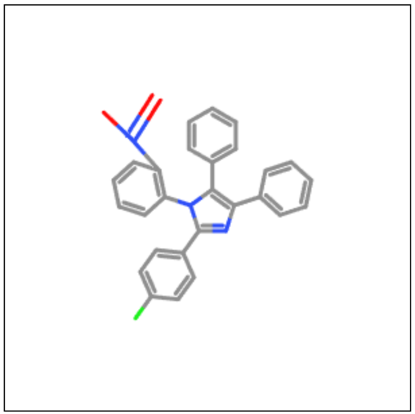

Predicted LD50: 938mg/kg

Predicted Toxicity Class: 4

1 2 3 4 5 6

Average similarity: 47.01%

Prediction accuracy: 54.26%

20% 40% 60% 80%

Name	
Molweight	451.9
Number of hydrogen bond acceptors	2
Number of hydrogen bond donors	0
Number of atoms	33
Number of bonds	37
Number of rotable bonds	5
Molecular refractivity	133.7
Topological Polar Surface Area	63.64
octanol/water partition coefficient(logP)	7.96

Toxicity Model Report

Copy Excel CSV PDF

Classification	Target	Shorthand	Prediction	Probability
Organ toxicity	Hepatotoxicity	dili	**Active**	0.53
Organ toxicity	Neurotoxicity	neuro	**Active**	0.59
Organ toxicity	Nephrotoxicity	nephro	Inactive	0.67
Organ toxicity	Respiratory toxicity	respi	**Active**	0.55
Organ toxicity	Cardiotoxicity	cardio	Inactive	0.73
Toxicity end points	Carcinogenicity	carcino	**Active**	0.68
Toxicity end points	Immunotoxicity	immuno	Inactive	0.92
Toxicity end points	Mutagenicity	mutagen	**Active**	0.68
Toxicity end points	Cytotoxicity	cyto	Inactive	0.71
Toxicity end points	BBB-barrier	bbb	**Active**	0.89
Toxicity end points	Ecotoxicity	eco	**Active**	0.79
Toxicity end points	Clinical toxicity	clinical	Inactive	0.54
Toxicity end points	Nutritional toxicity	nutri	Inactive	0.65
Tox21-Nuclear receptor signalling pathways	Aryl hydrocarbon Receptor (AhR)	nr_ahr	Inactive	0.62
Tox21-Nuclear receptor signalling pathways	Androgen Receptor (AR)	nr_ar	Inactive	0.98
Tox21-Nuclear receptor signalling pathways	Androgen Receptor Ligand Binding Domain (AR-LBD)	nr_ar_lbd	Inactive	0.93
Tox21-Nuclear receptor signalling pathways	Aromatase	nr_aromatase	**Active**	0.64
Tox21-Nuclear receptor signalling pathways	Estrogen Receptor Alpha (ER)	nr_er	Inactive	0.85
Tox21-Nuclear receptor signalling pathways	Estrogen Receptor Ligand Binding Domain (ER-LBD)	nr_er_lbd	Inactive	0.68
Tox21-Nuclear receptor signalling pathways	Peroxisome Proliferator Activated Receptor Gamma (PPAR-Gamma)	nr_ppar_gamma	Inactive	0.94
Tox21-Stress response pathways	Nuclear factor (erythroid-derived 2)-like 2/antioxidant responsive element (nrf2/ARE)	sr_are	Inactive	0.64
Tox21-Stress response pathways	Heat shock factor response element (HSE)	sr_hse	Inactive	0.64
Tox21-Stress response pathways	Mitochondrial Membrane Potential (MMP)	sr_mmp	Inactive	0.52
Tox21-Stress response pathways	Phosphoprotein (Tumor Supressor) p53	sr_p53	Inactive	0.75
Tox21-Stress response pathways	ATPase family AAA domain-containing protein 5 (ATAD5)	sr_atad5	Inactive	0.85
Molecular Initiating Events	Thyroid hormone receptor alpha (THRα)	mie_thr_alpha	Inactive	0.90
Molecular Initiating Events	Thyroid hormone receptor beta (THRβ)	mie_thr_beta	Inactive	0.78
Molecular Initiating Events	Transtyretrin (TTR)	mie_ttr	Inactive	0.97
Molecular Initiating Events	Ryanodine receptor (RYR)	mie_ryr	Inactive	0.98
Molecular Initiating Events	GABA receptor (GABAR)	mie_gabar	Inactive	0.96
Molecular Initiating Events	Glutamate N-methyl-D-aspartate receptor (NMDAR)	mie_nmdar	Inactive	0.92
Molecular Initiating Events	alpha-amino-3-hydroxy-5-methyl-4-isoxazolepropionate receptor (AMPAR)	mie_ampar	Inactive	0.97

Classification	Target	Shorthand	Prediction	Probability
Molecular Initiating Events	Kainate receptor (KAR)	mie_kar	Inactive	0.99
Molecular Initiating Events	Achetylcholinesterase (AChE)	mie_ache	**Active**	0.53
Molecular Initiating Events	Constitutive androstane receptor (CAR)	mie_car	Inactive	0.98
Molecular Initiating Events	Pregnane X receptor (PXR)	mie_pxr	Inactive	0.92
Molecular Initiating Events	NADH-quinone oxidoreductase (NADHOX)	mie_nadhox	Inactive	0.97
Molecular Initiating Events	Voltage gated sodium channel (VGSC)	mie_vgsc	Inactive	0.95
Molecular Initiating Events	Na+/I- symporter (NIS)	mie_nis	Inactive	0.98
Metabolism	Cytochrome CYP1A2	CYP1A2	**Active**	0.63
Metabolism	Cytochrome CYP2C19	CYP2C19	Inactive	0.51
Metabolism	Cytochrome CYP2C9	CYP2C9	Inactive	0.54
Metabolism	Cytochrome CYP2D6	CYP2D6	Inactive	0.64
Metabolism	Cytochrome CYP3A4	CYP3A4	**Active**	0.64
Metabolism	Cytochrome CYP2E1	CYP2E1	Inactive	0.99

Toxicity targets

Possible binding to toxicity targets is shown below. For more information on the targets, please click on the individual abbreviations.

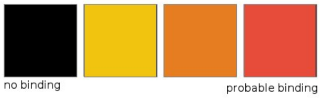

AA2AR	ADRB2	ANDR	AOFA	CRFR1	DRD3	ESR1	ESR2	GCR	HRH1	NR1I2	OPRK	OPRM	PDE4D	PGH1	PRGR

Details about possible toxicity targets:

	Toxicity Target	Avg Pharmacophore Fit	Avg Similarity Known Ligands
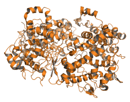	Prostaglandin G/H Synthase 1	0%	71.21%

Oral toxicity prediction results for input compound

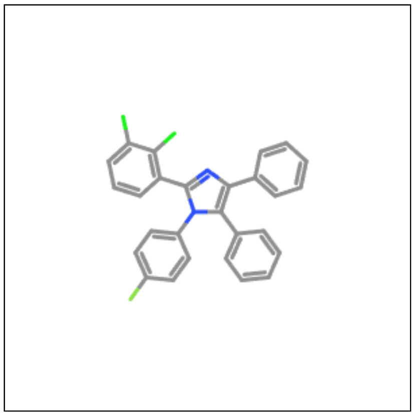

Predicted LD50: 938mg/kg

Predicted Toxicity Class: 4

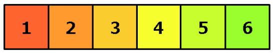

Average similarity: 48.03%

Prediction accuracy: 54.26%

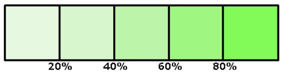

Name	
Molweight	459.34
Number of hydrogen bond acceptors	1
Number of hydrogen bond donors	0
Number of atoms	32
Number of bonds	36
Number of rotable bonds	4
Molecular refractivity	129.85
Topological Polar Surface Area	17.82
octanol/water partition coefficient(logP)	8.32

Toxicity Model Report

Copy Excel CSV PDF

Classification	Target	Shorthand	Prediction	Probability
Organ toxicity	Hepatotoxicity	dili	**Active**	0.51
Organ toxicity	Neurotoxicity	neuro	**Active**	0.89
Organ toxicity	Nephrotoxicity	nephro	Inactive	0.87
Organ toxicity	Respiratory toxicity	respi	**Active**	0.59
Organ toxicity	Cardiotoxicity	cardio	Inactive	0.91
Toxicity end points	Carcinogenicity	carcino	**Active**	0.52
Toxicity end points	Immunotoxicity	immuno	Inactive	0.90
Toxicity end points	Mutagenicity	mutagen	Inactive	0.61
Toxicity end points	Cytotoxicity	cyto	Inactive	0.87
Toxicity end points	BBB-barrier	bbb	**Active**	0.92
Toxicity end points	Ecotoxicity	eco	**Active**	0.77
Toxicity end points	Clinical toxicity	clinical	**Active**	0.64
Toxicity end points	Nutritional toxicity	nutri	Inactive	0.65
Tox21-Nuclear receptor signalling pathways	Aryl hydrocarbon Receptor (AhR)	nr_ahr	Inactive	0.54
Tox21-Nuclear receptor signalling pathways	Androgen Receptor (AR)	nr_ar	Inactive	0.97
Tox21-Nuclear receptor signalling pathways	Androgen Receptor Ligand Binding Domain (AR-LBD)	nr_ar_lbd	Inactive	0.99
Tox21-Nuclear receptor signalling pathways	Aromatase	nr_aromatase	**Active**	0.70
Tox21-Nuclear receptor signalling pathways	Estrogen Receptor Alpha (ER)	nr_er	Inactive	0.78
Tox21-Nuclear receptor signalling pathways	Estrogen Receptor Ligand Binding Domain (ER-LBD)	nr_er_lbd	Inactive	0.88
Tox21-Nuclear receptor signalling pathways	Peroxisome Proliferator Activated Receptor Gamma (PPAR-Gamma)	nr_ppar_gamma	Inactive	0.96
Tox21-Stress response pathways	Nuclear factor (erythroid-derived 2)-like 2/antioxidant responsive element (nrf2/ARE)	sr_are	Inactive	0.57
Tox21-Stress response pathways	Heat shock factor response element (HSE)	sr_hse	Inactive	0.57
Tox21-Stress response pathways	Mitochondrial Membrane Potential (MMP)	sr_mmp	Inactive	0.70
Tox21-Stress response pathways	Phosphoprotein (Tumor Supressor) p53	sr_p53	Inactive	0.82
Tox21-Stress response pathways	ATPase family AAA domain-containing protein 5 (ATAD5)	sr_atad5	Inactive	0.81
Molecular Initiating Events	Thyroid hormone receptor alpha (THRα)	mie_thr_alpha	Inactive	0.90
Molecular Initiating Events	Thyroid hormone receptor beta (THRβ)	mie_thr_beta	Inactive	0.78
Molecular Initiating Events	Transtyretrin (TTR)	mie_ttr	Inactive	0.97
Molecular Initiating Events	Ryanodine receptor (RYR)	mie_ryr	Inactive	0.98
Molecular Initiating Events	GABA receptor (GABAR)	mie_gabar	Inactive	0.96
Molecular Initiating Events	Glutamate N-methyl-D-aspartate receptor (NMDAR)	mie_nmdar	Inactive	0.92
Molecular Initiating Events	alpha-amino-3-hydroxy-5-methyl-4-isoxazolepropionate receptor (AMPAR)	mie_ampar	Inactive	0.97

Classification	Target	Shorthand	Prediction	Probability
Molecular Initiating Events	Kainate receptor (KAR)	mie_kar	Inactive	0.99
Molecular Initiating Events	Achetylcholinesterase (AChE)	mie_ache	**Active**	0.51
Molecular Initiating Events	Constitutive androstane receptor (CAR)	mie_car	Inactive	0.98
Molecular Initiating Events	Pregnane X receptor (PXR)	mie_pxr	Inactive	0.92
Molecular Initiating Events	NADH-quinone oxidoreductase (NADHOX)	mie_nadhox	Inactive	0.97
Molecular Initiating Events	Voltage gated sodium channel (VGSC)	mie_vgsc	Inactive	0.95
Molecular Initiating Events	Na+/I- symporter (NIS)	mie_nis	Inactive	0.98
Metabolism	Cytochrome CYP1A2	CYP1A2	**Active**	0.82
Metabolism	Cytochrome CYP2C19	CYP2C19	**Active**	0.80
Metabolism	Cytochrome CYP2C9	CYP2C9	**Active**	0.53
Metabolism	Cytochrome CYP2D6	CYP2D6	**Active**	0.82
Metabolism	Cytochrome CYP3A4	CYP3A4	**Active**	0.90
Metabolism	Cytochrome CYP2E1	CYP2E1	Inactive	0.96

Toxicity targets

Possible binding to toxicity targets is shown below. For more information on the targets, please click on the individual abbreviations.

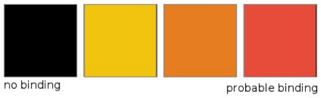

AA2AR	ADRB2	ANDR	AOFA	CRFR1	DRD3	ESR1	ESR2	GCR	HRH1	NR1I2	OPRK	OPRM	PDE4D	PGH1	PRGR

Details about possible toxicity targets:

	Toxicity Target	Avg Pharmacophore Fit	Avg Similarity Known Ligands
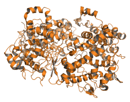	Prostaglandin G/H Synthase 1	0%	74.32%

Oral toxicity prediction results for input compound

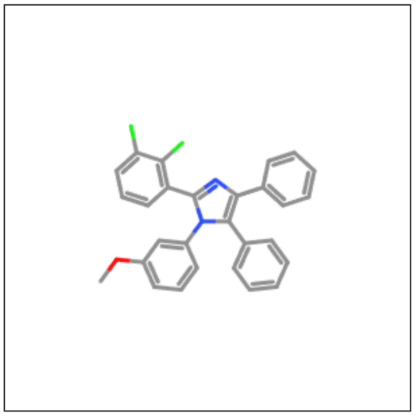

Predicted LD50: 2000mg/kg

Predicted Toxicity Class: 4

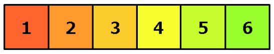

Average similarity: 53.34%

Prediction accuracy: 67.38%

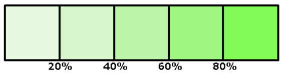

Name	
Molweight	471.38
Number of hydrogen bond acceptors	2
Number of hydrogen bond donors	0
Number of atoms	33
Number of bonds	37
Number of rotable bonds	5
Molecular refractivity	136.38
Topological Polar Surface Area	27.05
octanol/water partition coefficient(logP)	8.19

Toxicity Model Report

Copy Excel CSV PDF

Classification	Target	Shorthand	Prediction	Probability
Organ toxicity	Hepatotoxicity	dili	**Active**	0.56
Organ toxicity	Neurotoxicity	neuro	**Active**	0.84
Organ toxicity	Nephrotoxicity	nephro	Inactive	0.72
Organ toxicity	Respiratory toxicity	respi	**Active**	0.59
Organ toxicity	Cardiotoxicity	cardio	Inactive	0.84
Toxicity end points	Carcinogenicity	carcino	**Active**	0.54
Toxicity end points	Immunotoxicity	immuno	Inactive	0.62
Toxicity end points	Mutagenicity	mutagen	Inactive	0.54
Toxicity end points	Cytotoxicity	cyto	Inactive	0.59
Toxicity end points	BBB-barrier	bbb	**Active**	0.89
Toxicity end points	Ecotoxicity	eco	**Active**	0.76
Toxicity end points	Clinical toxicity	clinical	**Active**	0.67
Toxicity end points	Nutritional toxicity	nutri	Inactive	0.70
Tox21-Nuclear receptor signalling pathways	Aryl hydrocarbon Receptor (AhR)	nr_ahr	Inactive	0.57
Tox21-Nuclear receptor signalling pathways	Androgen Receptor (AR)	nr_ar	Inactive	0.96
Tox21-Nuclear receptor signalling pathways	Androgen Receptor Ligand Binding Domain (AR-LBD)	nr_ar_lbd	Inactive	0.99
Tox21-Nuclear receptor signalling pathways	Aromatase	nr_aromatase	**Active**	0.50
Tox21-Nuclear receptor signalling pathways	Estrogen Receptor Alpha (ER)	nr_er	Inactive	0.73
Tox21-Nuclear receptor signalling pathways	Estrogen Receptor Ligand Binding Domain (ER-LBD)	nr_er_lbd	Inactive	0.97
Tox21-Nuclear receptor signalling pathways	Peroxisome Proliferator Activated Receptor Gamma (PPAR-Gamma)	nr_ppar_gamma	Inactive	0.95
Tox21-Stress response pathways	Nuclear factor (erythroid-derived 2)-like 2/antioxidant responsive element (nrf2/ARE)	sr_are	Inactive	0.73
Tox21-Stress response pathways	Heat shock factor response element (HSE)	sr_hse	Inactive	0.73
Tox21-Stress response pathways	Mitochondrial Membrane Potential (MMP)	sr_mmp	Inactive	0.60
Tox21-Stress response pathways	Phosphoprotein (Tumor Supressor) p53	sr_p53	Inactive	0.85
Tox21-Stress response pathways	ATPase family AAA domain-containing protein 5 (ATAD5)	sr_atad5	Inactive	0.78
Molecular Initiating Events	Thyroid hormone receptor alpha (THRα)	mie_thr_alpha	Inactive	0.90
Molecular Initiating Events	Thyroid hormone receptor beta (THRβ)	mie_thr_beta	Inactive	0.78
Molecular Initiating Events	Transtyretrin (TTR)	mie_ttr	Inactive	0.97
Molecular Initiating Events	Ryanodine receptor (RYR)	mie_ryr	Inactive	0.98
Molecular Initiating Events	GABA receptor (GABAR)	mie_gabar	Inactive	0.96
Molecular Initiating Events	Glutamate N-methyl-D-aspartate receptor (NMDAR)	mie_nmdar	Inactive	0.92
Molecular Initiating Events	alpha-amino-3-hydroxy-5-methyl-4-isoxazolepropionate receptor (AMPAR)	mie_ampar	Inactive	0.97

Classification	Target	Shorthand	Prediction	Probability
Molecular Initiating Events	Kainate receptor (KAR)	mie_kar	Inactive	0.99
Molecular Initiating Events	Achetylcholinesterase (AChE)	mie_ache	**Active**	0.56
Molecular Initiating Events	Constitutive androstane receptor (CAR)	mie_car	Inactive	0.98
Molecular Initiating Events	Pregnane X receptor (PXR)	mie_pxr	Inactive	0.92
Molecular Initiating Events	NADH-quinone oxidoreductase (NADHOX)	mie_nadhox	Inactive	0.97
Molecular Initiating Events	Voltage gated sodium channel (VGSC)	mie_vgsc	Inactive	0.95
Molecular Initiating Events	Na+/I- symporter (NIS)	mie_nis	Inactive	0.98
Metabolism	Cytochrome CYP1A2	CYP1A2	**Active**	0.81
Metabolism	Cytochrome CYP2C19	CYP2C19	**Active**	0.55
Metabolism	Cytochrome CYP2C9	CYP2C9	Inactive	0.56
Metabolism	Cytochrome CYP2D6	CYP2D6	**Active**	0.71
Metabolism	Cytochrome CYP3A4	CYP3A4	**Active**	0.68
Metabolism	Cytochrome CYP2E1	CYP2E1	Inactive	0.99

Toxicity targets

Possible binding to toxicity targets is shown below. For more information on the targets, please click on the individual abbreviations.

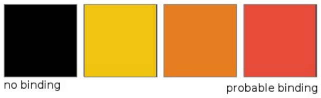

AA2AR	ADRB2	ANDR	AOFA	CRFR1	DRD3	ESR1	ESR2	GCR	HRH1	NR1I2	OPRK	OPRM	PDE4D	PGH1	PRGR

Details about possible toxicity targets:

	Toxicity Target	Avg Pharmacophore Fit	Avg Similarity Known Ligands
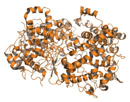	Prostaglandin G/H Synthase 1	0%	73.99%

Oral toxicity prediction results for input compound

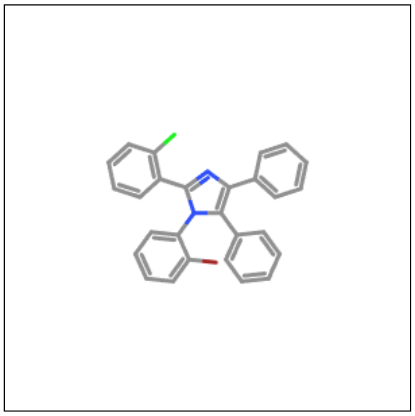

Predicted LD50: 2000mg/kg

Predicted Toxicity Class: 4

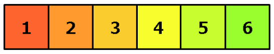

Average similarity: 48.64%

Prediction accuracy: 54.26%

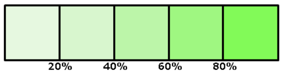

Name	
Molweight	485.8
Number of hydrogen bond acceptors	1
Number of hydrogen bond donors	0
Number of atoms	31
Number of bonds	35
Number of rotable bonds	4
Molecular refractivity	132.58
Topological Polar Surface Area	17.82
octanol/water partition coefficient(logP)	8.29

Toxicity Model Report

Copy Excel CSV PDF

Classification	Target	Shorthand	Prediction	Probability
Organ toxicity	Hepatotoxicity	dili	Inactive	0.50
Organ toxicity	Neurotoxicity	neuro	**Active**	0.89
Organ toxicity	Nephrotoxicity	nephro	Inactive	0.86
Organ toxicity	Respiratory toxicity	respi	**Active**	0.55
Organ toxicity	Cardiotoxicity	cardio	Inactive	0.90
Toxicity end points	Carcinogenicity	carcino	**Active**	0.54
Toxicity end points	Immunotoxicity	immuno	Inactive	0.98
Toxicity end points	Mutagenicity	mutagen	Inactive	0.61
Toxicity end points	Cytotoxicity	cyto	Inactive	0.83
Toxicity end points	BBB-barrier	bbb	**Active**	0.92
Toxicity end points	Ecotoxicity	eco	**Active**	0.76
Toxicity end points	Clinical toxicity	clinical	**Active**	0.63
Toxicity end points	Nutritional toxicity	nutri	Inactive	0.61
Tox21-Nuclear receptor signalling pathways	Aryl hydrocarbon Receptor (AhR)	nr_ahr	Inactive	0.51
Tox21-Nuclear receptor signalling pathways	Androgen Receptor (AR)	nr_ar	Inactive	0.97
Tox21-Nuclear receptor signalling pathways	Androgen Receptor Ligand Binding Domain (AR-LBD)	nr_ar_lbd	Inactive	0.99
Tox21-Nuclear receptor signalling pathways	Aromatase	nr_aromatase	**Active**	0.66
Tox21-Nuclear receptor signalling pathways	Estrogen Receptor Alpha (ER)	nr_er	Inactive	0.77
Tox21-Nuclear receptor signalling pathways	Estrogen Receptor Ligand Binding Domain (ER-LBD)	nr_er_lbd	Inactive	0.89
Tox21-Nuclear receptor signalling pathways	Peroxisome Proliferator Activated Receptor Gamma (PPAR-Gamma)	nr_ppar_gamma	Inactive	0.97
Tox21-Stress response pathways	Nuclear factor (erythroid-derived 2)-like 2/antioxidant responsive element (nrf2/ARE)	sr_are	Inactive	0.51
Tox21-Stress response pathways	Heat shock factor response element (HSE)	sr_hse	Inactive	0.51
Tox21-Stress response pathways	Mitochondrial Membrane Potential (MMP)	sr_mmp	Inactive	0.71
Tox21-Stress response pathways	Phosphoprotein (Tumor Supressor) p53	sr_p53	Inactive	0.80
Tox21-Stress response pathways	ATPase family AAA domain-containing protein 5 (ATAD5)	sr_atad5	Inactive	0.80
Molecular Initiating Events	Thyroid hormone receptor alpha (THRα)	mie_thr_alpha	Inactive	0.90
Molecular Initiating Events	Thyroid hormone receptor beta (THRβ)	mie_thr_beta	Inactive	0.78
Molecular Initiating Events	Transtyretrin (TTR)	mie_ttr	Inactive	0.97
Molecular Initiating Events	Ryanodine receptor (RYR)	mie_ryr	Inactive	0.98
Molecular Initiating Events	GABA receptor (GABAR)	mie_gabar	Inactive	0.96
Molecular Initiating Events	Glutamate N-methyl-D-aspartate receptor (NMDAR)	mie_nmdar	Inactive	0.92
Molecular Initiating Events	alpha-amino-3-hydroxy-5-methyl-4-isoxazolepropionate receptor (AMPAR)	mie_ampar	Inactive	0.97

Classification	Target	Shorthand	Prediction	Probability
Molecular Initiating Events	Kainate receptor (KAR)	mie_kar	Inactive	0.99
Molecular Initiating Events	Achetylcholinesterase (AChE)	mie_ache	Inactive	0.50
Molecular Initiating Events	Constitutive androstane receptor (CAR)	mie_car	Inactive	0.98
Molecular Initiating Events	Pregnane X receptor (PXR)	mie_pxr	Inactive	0.92
Molecular Initiating Events	NADH-quinone oxidoreductase (NADHOX)	mie_nadhox	Inactive	0.97
Molecular Initiating Events	Voltage gated sodium channel (VGSC)	mie_vgsc	Inactive	0.95
Molecular Initiating Events	Na+/I- symporter (NIS)	mie_nis	Inactive	0.98
Metabolism	Cytochrome CYP1A2	CYP1A2	**Active**	0.82
Metabolism	Cytochrome CYP2C19	CYP2C19	**Active**	0.92
Metabolism	Cytochrome CYP2C9	CYP2C9	**Active**	0.52
Metabolism	Cytochrome CYP2D6	CYP2D6	**Active**	0.83
Metabolism	Cytochrome CYP3A4	CYP3A4	**Active**	0.90
Metabolism	Cytochrome CYP2E1	CYP2E1	Inactive	0.96

Toxicity targets

Possible binding to toxicity targets is shown below. For more information on the targets, please click on the individual abbreviations.

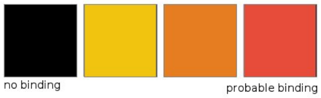

AA2AR	ADRB2	ANDR	AOFA	CRFR1	DRD3	ESR1	ESR2	GCR	HRH1	NR1I2	OPRK	OPRM	PDE4D	PGH1	PRGR

Details about possible toxicity targets:

	Toxicity Target	Avg Pharmacophore Fit	Avg Similarity Known Ligands
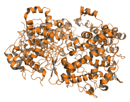	Prostaglandin G/H Synthase 1	0%	74.34%

Oral toxicity prediction results for input compound

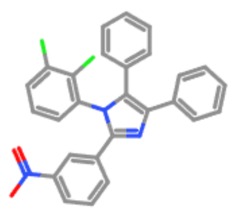

Predicted LD50: 500mg/kg

Predicted Toxicity Class: 4

1 2 3 4 5 6

Average similarity: 47.11%

Prediction accuracy: 54.26%

20% 40% 60% 80%

Name	
Molweight	486.35
Number of hydrogen bond acceptors	2
Number of hydrogen bond donors	0
Number of atoms	34
Number of bonds	38
Number of rotable bonds	5
Molecular refractivity	138.71
Topological Polar Surface Area	63.64
octanol/water partition coefficient(logP)	8.61

Toxicity Model Report

Copy Excel CSV PDF

Classification	Target	Shorthand	Prediction	Probability
Organ toxicity	Hepatotoxicity	dili	**Active**	0.52
Organ toxicity	Neurotoxicity	neuro	**Active**	0.60
Organ toxicity	Nephrotoxicity	nephro	Inactive	0.70
Organ toxicity	Respiratory toxicity	respi	**Active**	0.54
Organ toxicity	Cardiotoxicity	cardio	Inactive	0.70
Toxicity end points	Carcinogenicity	carcino	**Active**	0.66
Toxicity end points	Immunotoxicity	immuno	Inactive	0.90
Toxicity end points	Mutagenicity	mutagen	**Active**	0.64
Toxicity end points	Cytotoxicity	cyto	Inactive	0.69
Toxicity end points	BBB-barrier	bbb	**Active**	0.89
Toxicity end points	Ecotoxicity	eco	**Active**	0.79
Toxicity end points	Clinical toxicity	clinical	Inactive	0.53
Toxicity end points	Nutritional toxicity	nutri	Inactive	0.64
Tox21-Nuclear receptor signalling pathways	Aryl hydrocarbon Receptor (AhR)	nr_ahr	Inactive	0.63
Tox21-Nuclear receptor signalling pathways	Androgen Receptor (AR)	nr_ar	Inactive	0.98
Tox21-Nuclear receptor signalling pathways	Androgen Receptor Ligand Binding Domain (AR-LBD)	nr_ar_lbd	Inactive	0.91
Tox21-Nuclear receptor signalling pathways	Aromatase	nr_aromatase	**Active**	0.66
Tox21-Nuclear receptor signalling pathways	Estrogen Receptor Alpha (ER)	nr_er	Inactive	0.85
Tox21-Nuclear receptor signalling pathways	Estrogen Receptor Ligand Binding Domain (ER-LBD)	nr_er_lbd	Inactive	0.66
Tox21-Nuclear receptor signalling pathways	Peroxisome Proliferator Activated Receptor Gamma (PPAR-Gamma)	nr_ppar_gamma	Inactive	0.95
Tox21-Stress response pathways	Nuclear factor (erythroid-derived 2)-like 2/antioxidant responsive element (nrf2/ARE)	sr_are	Inactive	0.63
Tox21-Stress response pathways	Heat shock factor response element (HSE)	sr_hse	Inactive	0.63
Tox21-Stress response pathways	Mitochondrial Membrane Potential (MMP)	sr_mmp	**Active**	0.54
Tox21-Stress response pathways	Phosphoprotein (Tumor Supressor) p53	sr_p53	Inactive	0.78
Tox21-Stress response pathways	ATPase family AAA domain-containing protein 5 (ATAD5)	sr_atad5	Inactive	0.84
Molecular Initiating Events	Thyroid hormone receptor alpha (THRα)	mie_thr_alpha	Inactive	0.90
Molecular Initiating Events	Thyroid hormone receptor beta (THRβ)	mie_thr_beta	Inactive	0.78
Molecular Initiating Events	Transtyretrin (TTR)	mie_ttr	Inactive	0.97
Molecular Initiating Events	Ryanodine receptor (RYR)	mie_ryr	Inactive	0.98
Molecular Initiating Events	GABA receptor (GABAR)	mie_gabar	Inactive	0.96
Molecular Initiating Events	Glutamate N-methyl-D-aspartate receptor (NMDAR)	mie_nmdar	Inactive	0.92
Molecular Initiating Events	alpha-amino-3-hydroxy-5-methyl-4-isoxazolepropionate receptor (AMPAR)	mie_ampar	Inactive	0.97

Classification	Target	Shorthand	Prediction	Probability
Molecular Initiating Events	Kainate receptor (KAR)	mie_kar	Inactive	0.99
Molecular Initiating Events	Achetylcholinesterase (AChE)	mie_ache	**Active**	0.52
Molecular Initiating Events	Constitutive androstane receptor (CAR)	mie_car	Inactive	0.98
Molecular Initiating Events	Pregnane X receptor (PXR)	mie_pxr	Inactive	0.92
Molecular Initiating Events	NADH-quinone oxidoreductase (NADHOX)	mie_nadhox	Inactive	0.97
Molecular Initiating Events	Voltage gated sodium channel (VGSC)	mie_vgsc	Inactive	0.95
Molecular Initiating Events	Na+/I- symporter (NIS)	mie_nis	Inactive	0.98
Metabolism	Cytochrome CYP1A2	CYP1A2	**Active**	0.62
Metabolism	Cytochrome CYP2C19	CYP2C19	**Active**	0.53
Metabolism	Cytochrome CYP2C9	CYP2C9	Inactive	0.52
Metabolism	Cytochrome CYP2D6	CYP2D6	Inactive	0.61
Metabolism	Cytochrome CYP3A4	CYP3A4	**Active**	0.71
Metabolism	Cytochrome CYP2E1	CYP2E1	Inactive	0.99

Toxicity targets

Possible binding to toxicity targets is shown below. For more information on the targets, please click on the individual abbreviations.

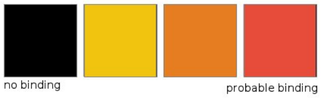

AA2AR	ADRB2	ANDR	AOFA	CRFR1	DRD3	ESR1	ESR2	GCR	HRH1	NR1I2	OPRK	OPRM	PDE4D	PGH1	PRGR

Oral toxicity prediction results for input compound

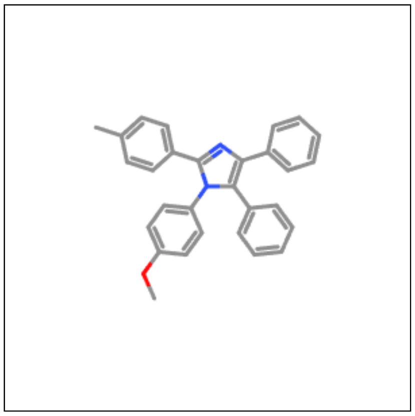

Predicted LD50: 2000mg/kg

Predicted Toxicity Class: 4

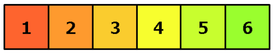

Average similarity: 57.8%

Prediction accuracy: 67.38%

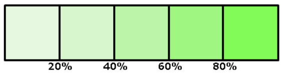

Name	
Molweight	416.51
Number of hydrogen bond acceptors	2
Number of hydrogen bond donors	0
Number of atoms	32
Number of bonds	36
Number of rotable bonds	5
Molecular refractivity	131.33
Topological Polar Surface Area	27.05
octanol/water partition coefficient(logP)	7.19

Toxicity Model Report

Copy Excel CSV PDF

Classification	Target	Shorthand	Prediction	Probability
Organ toxicity	Hepatotoxicity	dili	Inactive	0.56
Organ toxicity	Neurotoxicity	neuro	**Active**	0.81
Organ toxicity	Nephrotoxicity	nephro	Inactive	0.75
Organ toxicity	Respiratory toxicity	respi	**Active**	0.70
Organ toxicity	Cardiotoxicity	cardio	Inactive	0.81
Toxicity end points	Carcinogenicity	carcino	**Active**	0.57
Toxicity end points	Immunotoxicity	immuno	Inactive	0.99
Toxicity end points	Mutagenicity	mutagen	**Active**	0.56
Toxicity end points	Cytotoxicity	cyto	Inactive	0.63
Toxicity end points	BBB-barrier	bbb	**Active**	0.90
Toxicity end points	Ecotoxicity	eco	**Active**	0.67
Toxicity end points	Clinical toxicity	clinical	**Active**	0.65
Toxicity end points	Nutritional toxicity	nutri	Inactive	0.73
Tox21-Nuclear receptor signalling pathways	Aryl hydrocarbon Receptor (AhR)	nr_ahr	Inactive	0.56
Tox21-Nuclear receptor signalling pathways	Androgen Receptor (AR)	nr_ar	Inactive	0.96
Tox21-Nuclear receptor signalling pathways	Androgen Receptor Ligand Binding Domain (AR-LBD)	nr_ar_lbd	Inactive	0.98
Tox21-Nuclear receptor signalling pathways	Aromatase	nr_aromatase	Inactive	0.77
Tox21-Nuclear receptor signalling pathways	Estrogen Receptor Alpha (ER)	nr_er	Inactive	0.81
Tox21-Nuclear receptor signalling pathways	Estrogen Receptor Ligand Binding Domain (ER-LBD)	nr_er_lbd	Inactive	0.95
Tox21-Nuclear receptor signalling pathways	Peroxisome Proliferator Activated Receptor Gamma (PPAR-Gamma)	nr_ppar_gamma	Inactive	0.98
Tox21-Stress response pathways	Nuclear factor (erythroid-derived 2)-like 2/antioxidant responsive element (nrf2/ARE)	sr_are	Inactive	0.75
Tox21-Stress response pathways	Heat shock factor response element (HSE)	sr_hse	Inactive	0.75
Tox21-Stress response pathways	Mitochondrial Membrane Potential (MMP)	sr_mmp	Inactive	0.60
Tox21-Stress response pathways	Phosphoprotein (Tumor Supressor) p53	sr_p53	Inactive	0.84
Tox21-Stress response pathways	ATPase family AAA domain-containing protein 5 (ATAD5)	sr_atad5	Inactive	0.74
Molecular Initiating Events	Thyroid hormone receptor alpha (THRα)	mie_thr_alpha	Inactive	0.90
Molecular Initiating Events	Thyroid hormone receptor beta (THRβ)	mie_thr_beta	Inactive	0.78
Molecular Initiating Events	Transtyretrin (TTR)	mie_ttr	Inactive	0.97
Molecular Initiating Events	Ryanodine receptor (RYR)	mie_ryr	Inactive	0.98
Molecular Initiating Events	GABA receptor (GABAR)	mie_gabar	Inactive	0.96
Molecular Initiating Events	Glutamate N-methyl-D-aspartate receptor (NMDAR)	mie_nmdar	Inactive	0.92
Molecular Initiating Events	alpha-amino-3-hydroxy-5-methyl-4-isoxazolepropionate receptor (AMPAR)	mie_ampar	Inactive	0.97

Classification	Target	Shorthand	Prediction	Probability
Molecular Initiating Events	Kainate receptor (KAR)	mie_kar	Inactive	0.99
Molecular Initiating Events	Achetylcholinesterase (AChE)	mie_ache	Inactive	0.56
Molecular Initiating Events	Constitutive androstane receptor (CAR)	mie_car	Inactive	0.98
Molecular Initiating Events	Pregnane X receptor (PXR)	mie_pxr	Inactive	0.92
Molecular Initiating Events	NADH-quinone oxidoreductase (NADHOX)	mie_nadhox	Inactive	0.97
Molecular Initiating Events	Voltage gated sodium channel (VGSC)	mie_vgsc	Inactive	0.95
Molecular Initiating Events	Na+/I- symporter (NIS)	mie_nis	Inactive	0.98
Metabolism	Cytochrome CYP1A2	CYP1A2	**Active**	0.67
Metabolism	Cytochrome CYP2C19	CYP2C19	**Active**	0.78
Metabolism	Cytochrome CYP2C9	CYP2C9	Inactive	0.63
Metabolism	Cytochrome CYP2D6	CYP2D6	**Active**	0.73
Metabolism	Cytochrome CYP3A4	CYP3A4	**Active**	0.56
Metabolism	Cytochrome CYP2E1	CYP2E1	Inactive	0.97

Toxicity targets

Possible binding to toxicity targets is shown below. For more information on the targets, please click on the individual abbreviations.

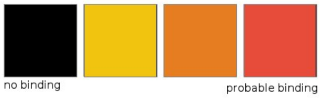

AA2AR	ADRB2	ANDR	AOFA	CRFR1	DRD3	ESR1	ESR2	GCR	HRH1	NR1I2	OPRK	OPRM	PDE4D	PGH1	PRGR

Details about possible toxicity targets:

	Toxicity Target	Avg Pharmacophore Fit	Avg Similarity Known Ligands
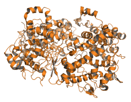	Prostaglandin G/H Synthase 1	0%	79.07%

Oral toxicity prediction results for input compound

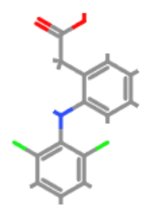

Predicted LD50: 53mg/kg

Predicted Toxicity Class: 3

1	2	3	4	5	6

Average similarity: 100%

Prediction accuracy: 100%

20% 40% 60% 80%

Name	
Molweight	296.15
Number of hydrogen bond acceptors	3
Number of hydrogen bond donors	2
Number of atoms	19
Number of bonds	20
Number of rotable bonds	4
Molecular refractivity	77.55
Topological Polar Surface Area	49.33
octanol/water partition coefficient(logP)	4.44

Toxicity Model Report

Copy Excel CSV PDF

Classification	Target	Shorthand	Prediction	Probability
Organ toxicity	Hepatotoxicity	dili	**Active**	0.81
Organ toxicity	Neurotoxicity	neuro	**Active**	0.75
Organ toxicity	Nephrotoxicity	nephro	**Active**	0.58
Organ toxicity	Respiratory toxicity	respi	**Active**	0.86
Organ toxicity	Cardiotoxicity	cardio	**Active**	0.52
Toxicity end points	Carcinogenicity	carcino	Inactive	0.64
Toxicity end points	Immunotoxicity	immuno	Inactive	0.99
Toxicity end points	Mutagenicity	mutagen	Inactive	0.78
Toxicity end points	Cytotoxicity	cyto	Inactive	0.74
Toxicity end points	BBB-barrier	bbb	**Active**	0.78
Toxicity end points	Ecotoxicity	eco	Inactive	0.50
Toxicity end points	Clinical toxicity	clinical	**Active**	0.61
Toxicity end points	Nutritional toxicity	nutri	Inactive	0.61
Tox21-Nuclear receptor signalling pathways	Aryl hydrocarbon Receptor (AhR)	nr_ahr	Inactive	0.64
Tox21-Nuclear receptor signalling pathways	Androgen Receptor (AR)	nr_ar	Inactive	0.99
Tox21-Nuclear receptor signalling pathways	Androgen Receptor Ligand Binding Domain (AR-LBD)	nr_ar_lbd	Inactive	0.99
Tox21-Nuclear receptor signalling pathways	Aromatase	nr_aromatase	Inactive	0.96
Tox21-Nuclear receptor signalling pathways	Estrogen Receptor Alpha (ER)	nr_er	Inactive	0.94
Tox21-Nuclear receptor signalling pathways	Estrogen Receptor Ligand Binding Domain (ER-LBD)	nr_er_lbd	Inactive	0.98
Tox21-Nuclear receptor signalling pathways	Peroxisome Proliferator Activated Receptor Gamma (PPAR-Gamma)	nr_ppar_gamma	**Active**	0.88
Tox21-Stress response pathways	Nuclear factor (erythroid-derived 2)-like 2/antioxidant responsive element (nrf2/ARE)	sr_are	Inactive	0.93
Tox21-Stress response pathways	Heat shock factor response element (HSE)	sr_hse	Inactive	0.93
Tox21-Stress response pathways	Mitochondrial Membrane Potential (MMP)	sr_mmp	Inactive	0.57
Tox21-Stress response pathways	Phosphoprotein (Tumor Supressor) p53	sr_p53	Inactive	0.71
Tox21-Stress response pathways	ATPase family AAA domain-containing protein 5 (ATAD5)	sr_atad5	Inactive	0.99
Molecular Initiating Events	Thyroid hormone receptor alpha (THRα)	mie_thr_alpha	Inactive	0.90
Molecular Initiating Events	Thyroid hormone receptor beta (THRβ)	mie_thr_beta	Inactive	0.78
Molecular Initiating Events	Transtyretrin (TTR)	mie_ttr	Inactive	0.97
Molecular Initiating Events	Ryanodine receptor (RYR)	mie_ryr	Inactive	0.98
Molecular Initiating Events	GABA receptor (GABAR)	mie_gabar	Inactive	0.96
Molecular Initiating Events	Glutamate N-methyl-D-aspartate receptor (NMDAR)	mie_nmdar	Inactive	0.92
Molecular Initiating Events	alpha-amino-3-hydroxy-5-methyl-4-isoxazolepropionate receptor (AMPAR)	mie_ampar	Inactive	0.97

Classification	Target	Shorthand	Prediction	Probability
Molecular Initiating Events	Kainate receptor (KAR)	mie_kar	Inactive	0.99
Molecular Initiating Events	Achetylcholinesterase (AChE)	mie_ache	**Active**	0.81
Molecular Initiating Events	Constitutive androstane receptor (CAR)	mie_car	Inactive	0.98
Molecular Initiating Events	Pregnane X receptor (PXR)	mie_pxr	Inactive	0.92
Molecular Initiating Events	NADH-quinone oxidoreductase (NADHOX)	mie_nadhox	Inactive	0.97
Molecular Initiating Events	Voltage gated sodium channel (VGSC)	mie_vgsc	Inactive	0.95
Molecular Initiating Events	Na+/I- symporter (NIS)	mie_nis	Inactive	0.98
Metabolism	Cytochrome CYP1A2	CYP1A2	Inactive	0.59
Metabolism	Cytochrome CYP2C19	CYP2C19	Inactive	0.90
Metabolism	Cytochrome CYP2C9	CYP2C9	**Active**	0.88
Metabolism	Cytochrome CYP2D6	CYP2D6	Inactive	0.89
Metabolism	Cytochrome CYP3A4	CYP3A4	Inactive	0.97
Metabolism	Cytochrome CYP2E1	CYP2E1	Inactive	1.0

Toxicity targets

Possible binding to toxicity targets is shown below. For more information on the targets, please click on the individual abbreviations.

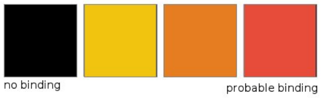

AA2AR	ADRB2	ANDR	AOFA	CRFR1	DRD3	ESR1	ESR2	GCR	HRH1	NR1I2	OPRK	OPRM	PDE4D	PGH1	PRGR

Details about possible toxicity targets:

	Toxicity Target	Avg Pharmacophore Fit	Avg Similarity Known Ligands
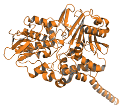	Amine Oxidase A	60.88%	0%
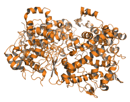	Prostaglandin G/H Synthase 1	62.82%	86.17%
